# Therapy and biomarker dependent progression-free survival in infant sonic hedgehog medulloblastoma: a multi-national retrospective cohort study

**DOI:** 10.1016/j.eclinm.2026.103913

**Published:** 2026-05-18

**Authors:** Stacey Richardson, Debbie Hicks, Melissa Gough, Ellie R. Butler, Dean Thompson, Jemma Castle, Stephen Crosier, Miguel Garcia-Ariza, Idoia Martín-Guerrero, Sabine L.A. Plasschaert, Franck Bourdeaut, Christelle Dufour, Julien Masliah-Planchon, Francesca Romana Buttarelli, Veronica Biassoni, Maura Massimino, Koichi Ichimura, Yonehiro Kanemura, Vijay Ramaswamy, Amar Gajjar, Marcel Kool, Andrey Korshunov, Stefan M. Pfister, Martin Mynarek, Stefan Rutkowski, Edward C. Schwalbe, Simon Bailey, Steven C. Clifford

**Affiliations:** a Wolfson Childhood Cancer Research Centre, Newcastle University Centre for Cancer, Translational and Clinical Research Institute, Newcastle University, Newcastle upon Tyne, UK; b Biobizkaia Health Research Institute, Pediatric Hematology and Oncology Unit, Hospital Universitario Cruces, Barakaldo, Spain; c Department of Genetics, Physical Anthropology & Animal Physiology, Biobizkaia Health Research Institute, University of the Basque Country, UPV/EHU, Leioa, Spain; d Princess Máxima Center for Pediatric Oncology, Utrecht, the Netherlands; e Paris-Cité University & SIREDO Oncology Center (Pediatric, Adolescent and Young Adults Oncology), Institut Curie, Paris, France; f Département de Cancérologie de l’Enfant et de l’Adolescent, Gustave Roussy, Paris, France; g Unité de Génétique Somatique, Institut Curie, Paris, France; h Dipartimento Scienze Oncologiche Radiologiche Anatomo-Patologiche, Sapienza Universita di Roma, Italy; i Dipartimento di Ematologia ed Oncoematologica Pediatrica, Fondazione IRCCS Istituto Nazionale dei Tumori di Milano, Milan, Italy; j Department of Pathology, Kyorin University Faculty of Medicine, Tokyo, Japan; k Department of Biomedical Research and Innovation, Institute for Clinical Research, NHO Osaka National Hospital, Osaka, Japan; l Department of Neurosurgery, NHO Osaka National Hospital, Osaka, Japan; m Programme in Developmental and Stem Cell Biology, The Hospital for Sick Children, Toronto, Ontario, Canada; n Department of Pediatric Medicine, St Jude Children’s Research Hospital, Memphis, TN, USA; o Division of Pediatric Neuro-Oncology (B062), Hopp Children’s Cancer Center Heidelberg (KiTZ), German Cancer Research Center (DKFZ), and German Cancer Consortium (DKTK), Heidelberg, Germany; p Clinical Cooperation Unit Neuropathology (B300), German Cancer Research Center (DKFZ), German Cancer Consortium (DKTK), and National Center for Tumor Diseases (NCT), Heidelberg, Germany; q Department of Pediatric Hematology and Oncology, Heidelberg University Hospital and National Center for Tumor Diseases (NCT), Heidelberg, Germany; r Department of Pediatric Hematology and Oncology, University Medical Center Hamburg-Eppendorf, Hamburg, Germany; s Department of Applied Sciences, Northumbria University, Newcastle upon Tyne, UK

**Keywords:** Medulloblastoma, Infant, SHH, Therapy, Prognosis, Biomarkers

## Abstract

**Background:**

Medulloblastoma in infants (iMB; aged < 5 years) presents the challenge of achieving cure while minimising deleterious cranio-spinal irradiation (CSI)-associated late-effects. Non-randomised phase 2 studies have examined upfront CSI omission and chemotherapy intensification for favourable-risk desmoplastic/nodular (DN) tumours associated with the sonic hedgehog (SHH) molecular group (iMB_SHH_). Comparison of these therapies in large molecularly defined iMB_SHH_ cohorts, alongside investigations of prognostic biomarkers in therapy-specific context, is urgently required to define future therapeutic strategies.

**Methods:**

In this international retrospective cohort study, a multi-national cohort of molecularly and clinically annotated iMB_SHH_ was assembled from patient datasets in nine countries. Inclusion criteria was a principal iMB_SHH_ group classification using DNA methylation array-based classification. Patient cohorts were assigned into upfront treatment groups based on the receipt of radiotherapy (RTx) or chemotherapy (CTx)-only. Upfront RTx treatment groups were assigned as those receiving focal-RTx or CSI. Upfront CTx only regimens used were classified into three groups to reflect disease treatment conventions: standard-dose, high-dose (intensified regimens of sufficient dosage to require stem cell support) and those including intraventricular methotrexate (IVT-MTX). We investigated molecular pathology, upfront treatments, and relationships to outcome, in this real-world setting. Outcomes of interest were progression-free survival (PFS), overall survival (OS), and post-relapse survival (PRS).

**Findings:**

Between January 20, 2018 and October 6, 2021, patient data from 267 infants with SHH medulloblastoma were collected from Canada (n = 74), Germany/USA (n = 67), and the UK (n = 54), alongside national cohorts collected from France (n = 26), Italy (n = 4), Japan (n = 20), the Netherlands (n = 11), and Spain (n = 33). 226 patients with PFS and OS data comprised the iMB survival cohort and were split into upfront treatment groups based on the receipt of RTx (n = 74, 33%) or CTx-only (n = 132, 58%). Among iMB_SHH_ patients treated upfront with CTx-only regimens, IVT-MTX therapy (5-year PFS, 72.6%; n = 72) or high-dose therapy (73.0%; n = 29) achieved PFS outcomes comparable to upfront CSI-based regimens (n = 49; 74.0%; p = 0.51); whereas lower-intensity, standard-dose, chemotherapy-only regimens (n = 31) were inferior (48.4% PFS; p = 0.006). Rescue was common post-relapse after IVT-MTX/high-dose protocols and translated into 5-year OS of 85.6% and 88.6%, respectively. However, information on pattern of relapse and treatments received at recurrence was only available for a small proportion of our cohort (n = 43). The 5-year PFS of patients receiving focal-RTx was (58.2%; n = 25). iMB_SHH_ encompassed SHH-1 (38%), SHH-2 (47%) and SHH-3 (14%) WHO subgroups. In CSI-naïve iMB_SHH_, standard-dose chemotherapy was associated with worse PFS in SHH-1 (p = 0.001), but not SHH-2. Non-DN/MBEN histology (21.2% of iMB_SHH_) conferred worse PFS in the upfront CSI-treated and standard-dose (p < 0.001 and p = 0.003, respectively) groups. Metastatic disease only associated with prognosis with upfront IVT-MTX-only therapies (p = 0.013), while established high-risk features of non-infant MB_SHH_ (*TP53*-mutation, LCA-histology, *MYCN-*amplification) only associated with poor prognosis in older SHH-3 (7/7 relapsed). Finally, CSI-naïve PFS findings were validated in a re-evaluation of smaller historical trials cohorts.

**Interpretation:**

Our findings show that iMB_SHH_ outcomes and prognostic biomarkers are therapy dependent. In our retrospective patient group, non-metastatic iMB_SHH_ treated with high-dose or IVT-MTX chemotherapy-only had equivalent favourable outcomes, independent of histology and subgroup. With outcomes established, clinical trials are now encouraged to focus on quality-of-life following different intensified approaches to identify the kindest curative strategies.

**Funding:**

10.13039/501100000289Cancer Research UK, 10.13039/501100001273Children with Cancer UK, Children’s Cancer North, Star for Harris, 10.13039/100010089JGW Patterson Foundation, Little Hero and Blue Skye Thinking.

**Research in context**

**Evidence before this study**

Around 30% of medulloblastomas arise in children under 5 years old (iMB) and present a major therapeutic challenge. Treatments aim to delay or avoid craniospinal irradiation (CSI) and limit its damaging life-long side-effects. However, these must be balanced with survival rates. Molecular biomarkers do not currently inform iMB therapies. We searched PubMed for articles published between January 1, 1990 and December 31, 2025, with the search terms “medulloblastoma” and “infant OR young” OR “trial”; 11 multi-institutional clinical trials were identified which enrolled iMB patients. To date, clinical studies (typically n < 50) have focussed on favourable-risk iMBs defined by DN/MBEN pathology (about 40% of iMB). However, while DN/MBEN are typically sonic hedgehog (SHH) molecular-group tumours, approximately 20% of infant SHH medulloblastomas (iMB_SHH_) are non-DN/MBEN; their clinical behaviour remains unclear. Previous studies have assessed upfront chemotherapy (CTx)-only approaches for DN/MBEN iMB, including standard-dose, high-dose or intraventricular methotrexate (IVT-MTX)-based regimens; studies have reported heterogeneous survival rates (31–93% 5-year PFS). In addition, iMB__SHH__ displays molecular heterogeneity (SHH-1, SHH-2, and more rarely SHH-3 (typical in older SHH) subgroups); the reported prognostic significance of these subgroups differs between studies.

**Added value of this study**

We assembled an unprecedented multi-centre cohort of molecularly defined iMB_SHH_ (n = 267) to directly compare outcomes and prognostic biomarkers for different upfront therapies across all clinico-pathological and molecular groups. Upfront high-dose and IVT-MTX based regimens produced progression-free survival (PFS) rates equivalent to CSI, supporting these intensified radiation-sparing approaches for this group. Moreover, prognostic relationships for clinico-molecular biomarkers are therapy-dependent; high-dose or IVT-MTX are required for better PFS in patients with SHH-1 tumours, while those with SHH-2 tumours have good outcomes independent of therapy. Exploratory analyses indicate metastatic disease only has prognostic significance in IVT-MTX-only therapies, and established high-risk biomarkers for non-infant MB_SHH_, are not relevant in SHH-1 and SHH-2. A combined reappraisal of previous trials concurred with our PFS findings.

**Implications of all the available evidence**

Our findings show that iMB_SHH_ survival outcomes and prognostic biomarkers are therapy dependent. This work is useful for extending and defining candidate groups for radiation-sparing; non-metastatic iMB_SHH_ has favourable outcomes, equivalent to CSI treatment, following upfront intensified high-dose or IVT-MTX chemotherapy, irrespective of the status of other putative high-risk features. Our study supports upcoming clinical trials in iMB, such as SIOP-CONNECT-COGNITO-MB (whose leadership includes authors of this study) and SJiMB21 (NCT05535166), which will focus on comparison of quality-of-life and neurocognitive impacts using these different intensified approaches, to identify least deleterious regimens.

## Introduction

Around 30% of medulloblastomas occur in infants and young children (iMB; aged under 3–5 years at diagnosis, depending on national treatment philosophy) and present an important paradigm for therapy de-intensification in paediatric oncology. Current therapeutic approaches in this age group differ from older children and aim to avoid or delay (to age 3–5 years) the use of craniospinal irradiation (CSI), to minimise damaging late-effects to the developing brain, and retain CSI for post-relapse rescue approaches.^1^, ^2^, ^3^ However, these strategies must be carefully balanced with curative intent.

iMB clinical trials to date have focussed on cohorts defined by desmoplastic nodular/medulloblastoma with extensive nodularity (DN/MBEN) or non-DN/MBEN disease pathology. The principal findings from these trials are that DN/MBEN tumours are more chemo-sensitive, can be rescued post-relapse, and display a more favourable prognosis. In contrast, outcomes for non-DN/MBEN iMB using radiation-sparing strategies are worse.^4^, ^5^, ^6^, ^7^, ^8^ Trials which have described avoidance of upfront CSI in DN/MBEN iMB have been modestly-sized (n < 42), and have employed specific chemotherapy (CTx) regimens (Supplementary Table S1)^4^, ^5^, ^6^, ^7^, ^8^ which can be classified into standard-dose, intraventricular-methotrexate (IVT-MTX) and high-dose (requiring stem-cell support) CTx-based protocols. Individually, they report different treatment responses and outcomes, and these different approaches have not been compared directly in larger or randomised studies. Similarly, metastatic disease may be an important prognostic factor; however, reports are inconclusive and experience is limited - most trials included few or no metastatic patients.^4^^,^^6^^,^^7^^,^^9^^,^^10^

Advances in medulloblastoma molecular pathology, including discovery of its four molecular groups (MB_WNT_, MB_SHH_, MB_Group3_ and MB_Group4_), including sonic hedgehog (SHH), have underpinned biomarker-directed clinical trials and risk-adapted therapies in non-infant MB.^11^^,^^12^ By contrast, prospective biomarkers have not yet informed eligibility or therapy assignment in iMB; however, a series of features show potential prognostic significance. Approximately 40% of iMB patients have MB_SHH_ tumours (iMB_SHH_), highly associated with DN/MBEN histology and distinct molecular features (e.g., *SUFU* mutations).^2^^,^^4^, ^5^, ^6^^,^^8^ However, while iMB DN/MBEN are typically MB_SHH_ tumours, ∼20% of iMB_SHH_ are non-DN/MBEN; these have not been captured alongside DN/MBEN cases in clinical trials and their clinical behaviour remains unclear, with a poorer prognosis indicated in some studies.^2^^,^^13^ Moreover, significant molecular diversity exists within iMB_SHH_. The 2021 WHO classification of brain tumours describes four MB_SHH_ subgroups (SHH-1-4).^14^ iMB_SHH_ predominantly consists of subgroups SHH-1 and SHH-2, with SHH-3 dominant in older children and SHH-4 in adults.^2^^,^^4^^,^^5^^,^^9^^,^^15^ iMB SHH-1 has been associated with poorer outcomes, but its significance differs between reported studies,^2^^,^^4^^,^^8^^,^^9^ suggesting potential therapy-dependency. Initial studies report post-relapse survival of iMB_SHH_ following failure of radiation-sparing approaches is approximately 60%, indicating cure at initial diagnosis is crucial.^16^^,^^17^ Finally, the relevance of poor-risk features established in older children with MB_SHH_ (e.g., *MYCN* amplification and *TP53* mutation in SHH-3), remains unclear in iMB_SHH_.

The evidence-based definition of most effective treatments, together with their associated responsive disease groups, is essential to optimally balance survival and survivorship outcomes for iMB_SHH_ patients. Comparative evaluations, in large molecularly-defined iMB_SHH_ cohorts, alongside investigations of prognostic biomarkers in therapy-specific context, are thus urgently required. However, no further clinical trials are expected to inform these issues in the short to medium-term.

To address this deficiency, we gathered the ‘real-world’ experience of iMB_SHH_ clinical behaviour and its relationship to therapy and biomarkers, based on an international molecularly and clinically annotated cohort of unprecedented scale encompassing all group demographics (n = 267). Through this analysis, alongside a re-evaluation of past clinical trials in the context of our findings, we resolve the biomarker and therapy-dependent nature of iMB_SHH_ survival outcomes. We define the therapies and favourable-risk disease groups essential to enable the next-generation of molecularly-driven trials, focussed on quality-of-life and neurocognitive impacts of effective therapies as their primary outcome measures, to identify kindest curative strategies.

## Methods

### Study design and participants

**Fig. 1:**
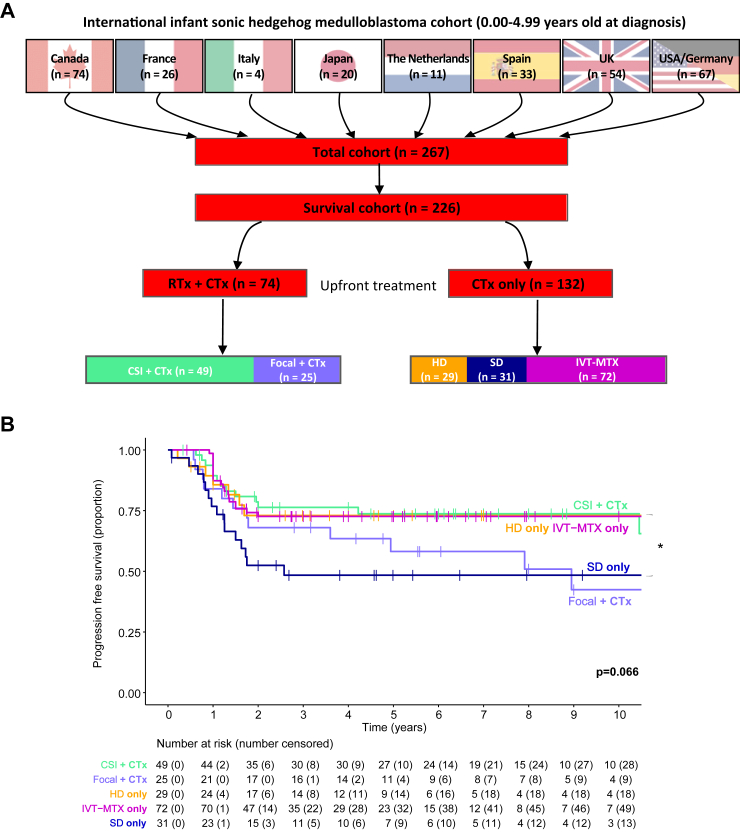
**A multi-national infant sonic hedgehog medulloblastoma (iMB_SHH_) cohort: Upfront treatments, and outcomes.** (A) Flowchart demonstrating patient samples and data collected in the total cohort, survival cohort, and upfront treatment groups. (B) Kaplan–Meier plot of progression-free survival for iMB_SHH_ upfront treatment groups: CSI + CTx, Focal + CTx, HD-CTx only, IVT-MTX only and SD-CTx only. At-risk tables (number censored in parentheses) and p-values from log-rank tests are shown. ∗Indicates p-value < 0.05. Red = iMB_SHH_, RTx = Radiotherapy. CTx = Chemotherapy. CSI = Craniospinal irradiation. SD = Standard-dose. HD = High-dose. IVT-MTX = Intraventricular methotrexate.

We assembled between 2018 and 2021 a retrospective multi-national cohort of 267 molecularly and clinically-annotated iMB_SHH_ (0.00–4.99 years of age diagnosis; Fig. 1A). An accompanying analysis of non-WNT/non-SHH iMB collected in parallel is reported elsewhere.^18^ The study size reflects all eligible cases identified across participating centres during the study period which met the inclusion criteria; no formal sample size calculation was performed as this was a retrospective cohort.

Inclusion criteria was a principal iMB_SHH_ group classification using DNA methylation array-based classification. Patient data were collected from international cohorts assembled in Canada,^15^ Germany/USA,^19^ and the UK,^2^ alongside national cohorts collected from France, Italy, Japan, the Netherlands, and Spain. To minimise selection and measurement bias across contributing centres, patient clinical and molecular data were collected from contributing institutions and centrally reviewed using standardised annotation protocols. Biological sex was taken from clinical records and confirmed using methylation array sex chromosome signal patterns. Patients have not been previously reported in clinical trials; outcomes of a subset of patients treated with IVT-MTX chemotherapy only have previously been reported^4^^,^^8^ and were included to enable the detailed analysis of this treatment group. Full demographic and clinical data, including treatment protocols, are given in Supplementary Table S2. Race and ethnicity data were not collected. Patients with progression-free survival (PFS) and overall survival (OS) data comprised the iMB survival cohort.

### Ethics

Ethical approval and written informed consents for data and sample collection were confirmed by each contributing group. Analyses were conducted with approval from Newcastle and North Tyneside Research Ethics Committee (study reference 07/Q0909/71).

### Procedures

All samples were assessed with Illumina HumanMethylation450K or EPIC DNA methylation array (Illumina, San Diego, CA, USA). Tumours were assigned to the SHH molecular subgroups (SHH-1, SHH-2, SHH-3, SHH-4) as previously described using ‘MNP Medulloblastoma classifier’ version 12.5 (www.molecularneuropathology.org/mnp) (confidence score >0.8).

We assessed established medulloblastoma clinical, pathological, and molecular features as described previously; institutional annotation was used. Histopathological variants were defined according to WHO 2016 guidelines.^20^ Metastatic status was assigned based on Chang’s criteria (M+; M stages 1–4, M0; local disease only).^21^ Tumours were designated sub-totally resected (STR) if their residuum after surgical excision was >1.5 cm^2^ and gross-totally resected (GTR) if < 1.5 cm^2^.^22^ Where available, we assessed recurrent MB mutations (*TP53*, *PTCH1*, *SUFU*, *KMT2D*, *SMO*; institutional annotation) and oncogene amplifications (*MYC*, *MYCN*). Tumour samples were analysed for chromosomal arm-level copy number changes in DNA methylation array datasets (Illumina, San Diego, CA, USA) using the R package ‘conumee’ (www.bioconductor.org/packages/conumee/) as previously described.^23^^,^^24^

Patient cohorts were assigned into upfront treatment groups based on the receipt of RTx or CTx-only; treatment selection was made at local treating centres according to local/national treatment philosophy. Upfront RTx treatment groups were assigned as focal-RTx (survival cohort; n = 25) or CSI (all regimens; n = 49). Upfront CTx only regimens used were classified into three groups; standard-dose (survival cohort; n = 31), high-dose (intensified regimens of sufficient dosage to require stem cell support; n = 29) and those including intraventricular methotrexate (IVT-MTX; n = 72) (Supplementary Table S2).

### Outcomes

Outcomes of interest were progression-free survival (PFS), overall survival (OS), and post-relapse survival (PRS). OS was defined as the interval between diagnosis and death or date of last follow-up. PFS was defined as the time from date of surgery to first event (progression or relapse) or date of last follow-up. Post-relapse survival (PRS) was defined as the time from first event to death or last follow-up.

### Statistical analysis

Associations between features were assessed by chi-squared and Fisher’s exact tests. The log-rank test was used in univariable analyses to assess OS, PFS and PRS across all groups and for all pairwise comparisons (only statistically significant pairwise comparisons are shown) within this study, and between this study and the Smith et al. (2025) study.^25^ The Kaplan–Meier method was used to visualise survival. Patients were censored at the date of last follow-up; the number censored is indicated in Kaplan–Meier at-risk tables. Groups with fewer than five patients were excluded from analyses shown in main figures. Univariable and multivariable Cox proportional hazards tests were used to investigate the association of features with survival, using forward likelihood-ratio testing (due to cohort size, multivariable testing was only performed for the upfront CTx-only iMB_SHH_ cohort). Multivariable Cox models were constructed using established clinical variables (metastatic status, extent of resection, histology) in addition to treatment and molecular factors (subgroup and CTx type). Age was analysed continuously and categorically (<3.00 versus ≥3.00 years) reflecting disease paradigms and the age-dependent threshold for use of radiotherapy. The assumptions for each statistical analysis were confirmed and p-values < 0.05 considered statistically significant. Analyses were complete-case; no imputation was performed.

Survival analysis was performed using the R package “survival” v3.4. Proportionality of hazards was assessed in Cox models using the ‘cox.zph’ function in the R package “survival” v3.4. Analyses and visualisation were performed in the R statistical environment (version 4.2.2). Percentages were calculated from samples with available data within the cohort and no statistical methods were used to predetermine cohort size.

No pre-planned sensitivity analyses were performed. Given the retrospective, multi-national design of this study and the challenges inherent in assembling molecularly annotated infant medulloblastoma cohorts of sufficient size, the primary analyses were designed to maximise the use of all available data meeting the pre-specified inclusion criteria. Comparison of the progression-free survival of this study and the Smith et al. study^25^ was performed post-hoc.

### Role of the funding source

The funders of the study had no role in study design, data collection, data analysis, data interpretation, or writing of the report. The corresponding author had full access to all of the data and had the final responsibility to submit for publication.

## Results

Between January 20, 2018 and October 6, 2021, patient data from 267 infants with SHH medulloblastoma were collected from international cohorts assembled in Canada (n = 74; 27.7%),^15^ Germany/USA (n = 67; 25.1%),^19^ and the UK (n = 54; 20.2%),^2^ alongside national cohorts collected from France (n = 26; 9.7%), Italy (n = 4; 1.5%), Japan (n = 20; 7.5%), the Netherlands (n = 11; 4.1%), and Spain (n = 33; 12.4%). Patients with progression-free survival (PFS) and overall survival (OS) data comprised the iMB survival cohort (n = 226). Patient within the survival cohort were assigned into upfront treatment groups based on the receipt of RTx (n = 74) or CTx-only (n = 132). Upfront RTx treatment groups were assigned as focal-RTx (n = 25) or CSI (all regimens; n = 49). Upfront CTx only regimens represented standard-dose therapy (n = 31), high-dose therapy (n = 29) and IVT-MTX (n = 72). Outcomes of a subset (n = 32, 44%) of patients treated with IVT-MTX chemotherapy only have previously been reported.^4^^,^^8^

We first examined outcomes associated with the different upfront therapeutic approaches employed for iMB_SHH_ across our cohort (Fig. 1A, Supplementary Table S2). 49 patients received CSI-based regimens and, as anticipated, were older than patients receiving radiation-sparing treatments (Supplementary Table S2). CSI outcomes were superior to focal irradiation-based therapies (n = 25) (5-year PFS; CSI 74%, 95% CI 62–88%, Focal 58%, 95% CI 41–82%, p = 0.11) (Supplementary Table S2), and comparable to those reported for non-infant MB_SHH_ (where standard therapy includes upfront CSI; 5-year PFS 58–74%, 95% CI 49–88%) (Supplementary Fig. S1).^24^^,^^25^ As anticipated, iMB_SHH_ patients who relapsed following upfront CSI were not commonly rescued (Supplementary Fig. S2C).

The majority of iMB_SHH_ patients received upfront CTx-only treatments (58.4%, 132/226) and were typically younger than 3 years at diagnosis (p < 0.001, Supplementary Fig. S2B). CTx-only regimens were classified into three groups to reflect disease treatment conventions: standard-dose (n = 31), high-dose (n = 29), and those including intraventricular methotrexate (IVT-MTX, n = 72) (Fig. 1, Supplementary Fig. S2A). Cohort demographics for the three groups are shown in Supplementary Table S2.

Patients treated with upfront high-dose-CTx (5-year PFS 73%, 95% CI 58–93%) or IVT-MTX (5-year PFS 73%, 95% CI 63–84%) had equivalent 5-year PFS, comparable to CSI-based therapy, and significantly superior to standard-dose-CTx regimens (p = 0.006, Fig. 1B, Supplementary Table S2). Post-relapse rescue was achievable following upfront CTx-only (5-year post-relapse survival (PRS) 48% overall, 95% CI 34–69%); PRS outcomes were equivalent and independent of upfront chemotherapy received (Supplementary Figs. S2C and 3A–E). These translated into respective 5-year overall survival (OS) rates of 88.6% (95% CI 77–100%) and 85.6% (95% CI 77–96%) for patients treated upfront with high-dose-CTx and IVT-MTX respectively, significantly superior to standard-dose regimens (p = 0.027) (Supplementary Fig. S2D).

**Fig. 2:**
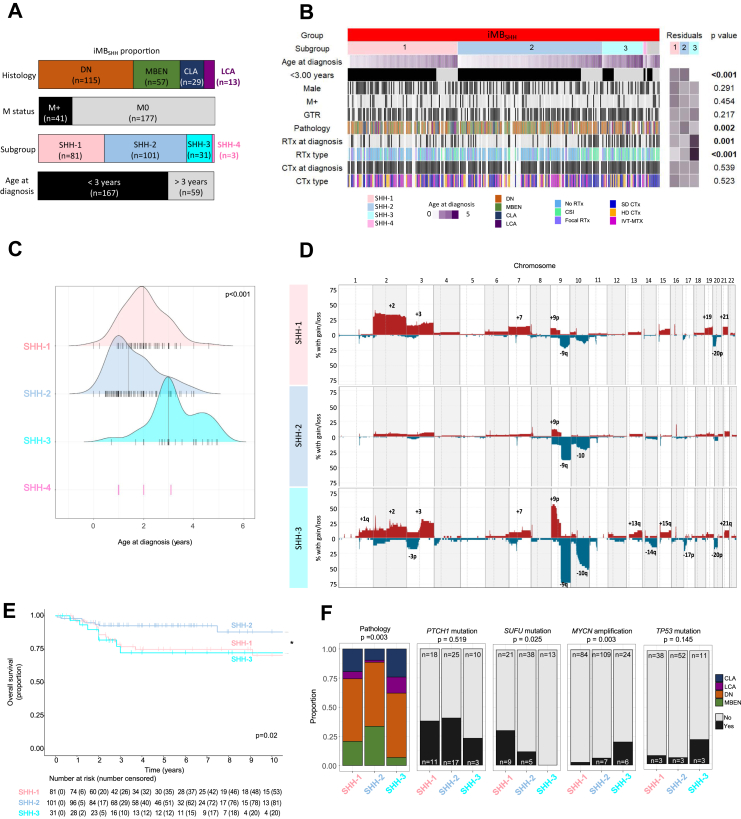
**Molecular and clinical diversity within iMB_SHH_ and characteristics of its molecular subgroups.** (A) Proportion and incidence of histology variants, metastatic (M) status, molecular subgroup and age at diagnosis. (B) Oncoplot representing key clinico-pathological and molecular disease features in iMB_SHH_ subgroups. Residuals and p-values from chi-squared tests are shown (darker shades indicate stronger relationships). (C) Density plot showing incidence of iMB_SHH_ subgroups across age range 0.00–4.99 years. (D) Summary of chromosomal copy number changes in iMB_SHH_ subgroups. Red = copy number gain. Blue = copy number loss. (E) Kaplan–Meier plot of overall survival for iMB_SHH_ subgroups. At-risk table (number censored in parentheses) and p-values from log-rank tests. ∗Indicates p-value < 0.05. (F) Stacked bar charts showing proportions of histological subtypes, *PTCH1* mutation, *SUFU* mutation, *MYCN* amplification and *TP53* mutation across iMB_SHH_ subgroups. p-values from Chi-squared tests are shown. Dark grey bar = positive for feature. Light grey bar = negative for feature. RTx = Radiotherapy. CTx = Chemotherapy. CSI = Craniospinal irradiation. SD = Standard-dose. HD = High-dose. IVT-MTX = Intraventricular methotrexate. CLA = Classic histology. LCA = Large-cell/anaplastic histology. MBEN = Medulloblastoma with extensive nodularity. DN = Desmoplastic nodular. GTR = Cross-total resection. M+ = Metastatic disease. M0 = Non-metastatic disease.

Molecular and pathological assessment of our cohort characterised the significant inter-patient diversity within iMB_SHH_ (Fig. 2 and Supplementary Fig. S4); 21.2% (50/136) of tumours had non-DN/MBEN pathology and 19.2% (44/229) were metastatic (Fig. 2A and B). Characterisation of WHO molecular subgroups revealed the expected predominance of SHH-1 (81/216, 37.5%) and SHH-2 (101/216, 46.8%) tumours.^15^^,^^26^ There were fewer SHH-3 (31/216, 14.4%), consistent with their older age at diagnosis (median age 3.0 years versus SHH-1 (2.0 years) and SHH-2 (1.4 years)) (Fig. 2C). Only 3 patients had SHH-4 subgroup tumours (age 1.0, 2.0 and 3.1 years at diagnosis). SHH-2 commonly displayed quiet copy number profiles, with only chromosome 9p gain and losses of chromosome 9q and 10 occurring frequently. Conversely, SHH-1 and SHH-3 frequently displayed multiple copy number changes including gains of chromosomes 2, 3 and 7 (Fig. 2D); gains of chromosome 19 and losses of 20p distinguished SHH-1 from the SHH-2 subgroups. Overall, considering all treatment groups together, patients with SHH-2 tumours had significantly better survival outcomes (5-year OS 93%, 95% CI 87–98%) than SHH-1 (5-year OS 75%, 95% CI 65–87%) and SHH-3 (5-year OS 72%, 95% CI 56–93%) (Fig. 2E). The frequencies of histological variants, *MYCN* amplification and *SUFU* mutation varied significantly between SHH subgroups (Fig. 2F).

**Fig. 3:**
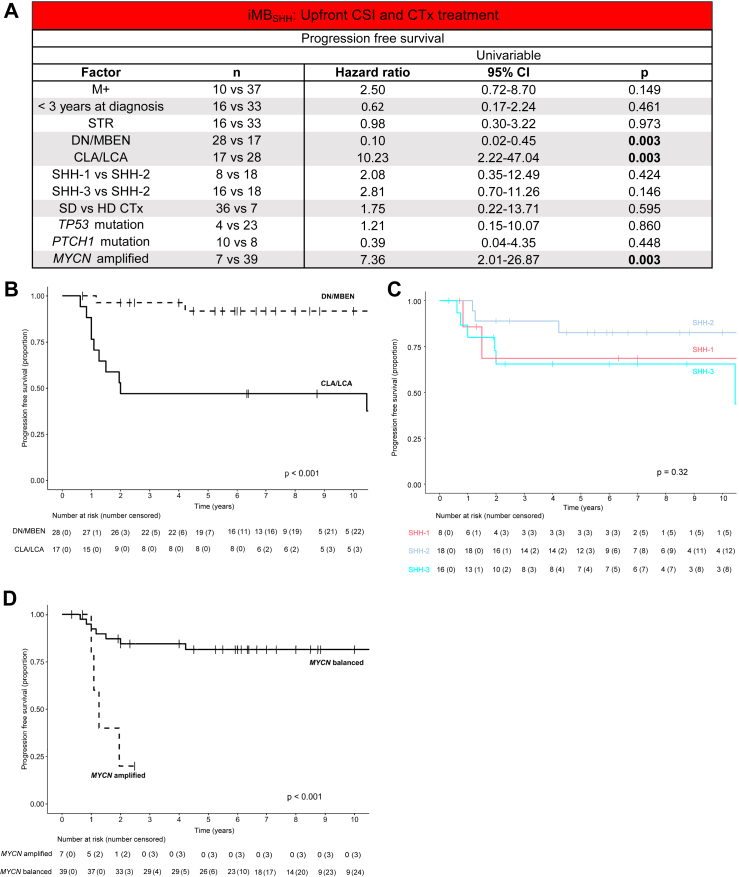
**Progression-free survival of iMB_SHH_ patients receiving upfront CSI and CTx treatment.** (A) Univariable Cox proportional hazards regression model of progression-free survival. p-values for Cox proportional hazards tests are shown. (B) Kaplan–Meier plot of progression-free survival according to DN/MBEN versus CLA/LCA pathological variants. At-risk tables (numbers censored in parentheses) and p-values from log-rank tests are shown. (C) Kaplan–Meier plot of progression-free survival according to MYCN amplification status. At-risk tables (numbers censored in parentheses) and p-values from log-rank tests are shown. (D) Kaplan–Meier plot of progression-free survival according to SHH molecular subgroup. At-risk tables (numbers censored in parentheses) and p-values from log-rank tests are shown. CTx = Chemotherapy. CSI = Craniospinal irradiation. SD = Standard-dose. HD = High-dose. STR = Sub-total resection. M+ = Metastatic disease. DN = Desmoplastic nodular. MBEN = Medulloblastoma with extensive nodularity. CLA = Classic histology. LCA = Large cell anaplastic histology. CI = Confidence interval.

We next investigated putative prognostic factors within iMB_SHH_ in the context of specific therapies received, to assess any treatment-dependence in their clinical behaviour. Univariable Cox regression analyses of patients treated with upfront CSI (Fig. 3A) identified a significant association between non-DN/MBEN histology and worse PFS (DN/MBEN HR 0.10, CI 0.02–0.45, p = 0.003) (Fig. 3A and B). Furthermore, *MYCN* amplification conferred worse survival (HR 7.36, CI 2.01–26.87, p = 0.003) (Fig. 3A, D). The SHH-1 and SHH-2 subgroups had equivalent outcomes (Fig. 3C). Equivalent relationships were observed for these factors against OS (Supplementary Fig. S5). Multivariable analysis was not performed in this group, due to cohort size limitations.

**Fig. 4:**
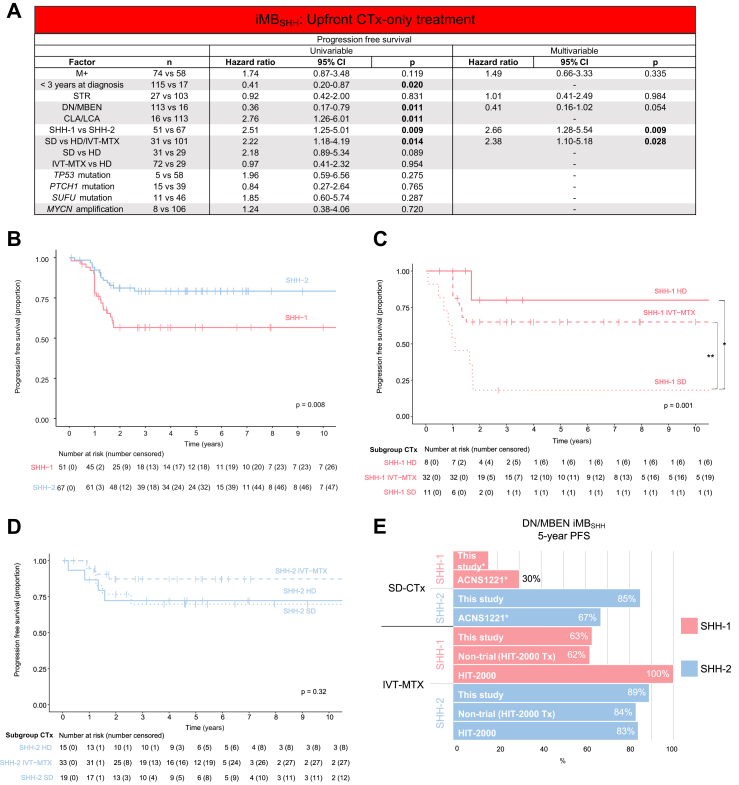
**Progression-free survival of iMB_SHH_ patients receiving upfront CTx-only treatment.** (A) Univariable and multivariable Cox proportional hazards regression model of progression-free survival. p-values for Cox proportional hazards tests are shown. (B) Kaplan–Meier plot of progression-free survival according to SHH molecular subgroup. At-risk table (numbers censored in parentheses) and p-value for log-rank test are shown. (C) Kaplan–Meier plot of progression-free survival for iMB SHH-1 according to CTx type. At-risk table (numbers censored in parentheses) and p-value for log-rank test are shown. ∗ Indicates p-value < 0.05. ∗∗ p-value 0.01–0.05. (D) Kaplan–Meier plot of progression-free survival for iMB SHH-2 according to CTx type. At-risk table (numbers censored in parentheses) and p-value for log-rank test are shown. (E) Mean 5-year progression-free survival proportion of previous DN/MBEN iMB_SHH_ studies according to SHH molecular subgroups and upfront CTx type. Figure includes previous iMB studies for which PFS information could be extracted for comparable patient cohorts. ∗ACNS1221 study reported 2-year PFS. CTx = Chemotherapy. STR = Sub-total resection. M+ = Metastatic disease. CLA = Classic histology. LCA = Large-cell/anaplastic histology. MBEN = Medulloblastoma with extensive nodularity. DN = Desmoplastic nodular. SD = Standard-dose. HD = High-dose. IVT-MTX = Intraventricular methotrexate. CI = Confidence interval. PFS = Progression-free survival.

Focussing on patients receiving upfront CTx-only, univariable analysis revealed both DN/MBEN histology and SHH-2 were associated with improved PFS (DN/MBEN HR 0.36, CI 0.17–0.79, p = 0.011; SHH-1 versus SHH-2 HR 2.51, CI 1.25–5.01, p = 0.009), and standard-dose-CTx was associated with poorer PFS (standard-dose versus high-dose/IVT-MTX HR 2.22, CI 1.18–4.19, p = 0.014) (Fig. 4A–C). Molecular subgroup and CTx type were the independent prognostic risk factors in multivariable analysis (SHH-1 HR 2.66, CI 1.28–5.54, p = 0.009, standard-dose versus high-dose/IVT-MTX HR 2.38, CI 1.10–5.18, p = 0.028). Considering the interaction between SHH subgroup and CTx, outcomes for SHH-1 and SHH-2 were dependent on the upfront CTx regimen used (Fig. 4D; Supplementary Fig. S6A and B). Standard-dose therapy was associated with worse PFS than high-dose (p = 0.013) and IVT-MTX (p = 0.0014) in SHH-1 (p = 0.001), while all three therapy groups had equivalent PFS in SHH-2. Finally, the intimate association between DN/MBEN histology and iMB_SHH_ enables validation of our findings in previous trials in DN/MBEN iMB (Fig. 4E, Supplementary Table S1). The worse PFS outcomes observed for SHH-1 patients using standard-dose-CTx were validated in the ACNS1221 trial (2-year PFS; 30%).^11^

**Fig. 5:**
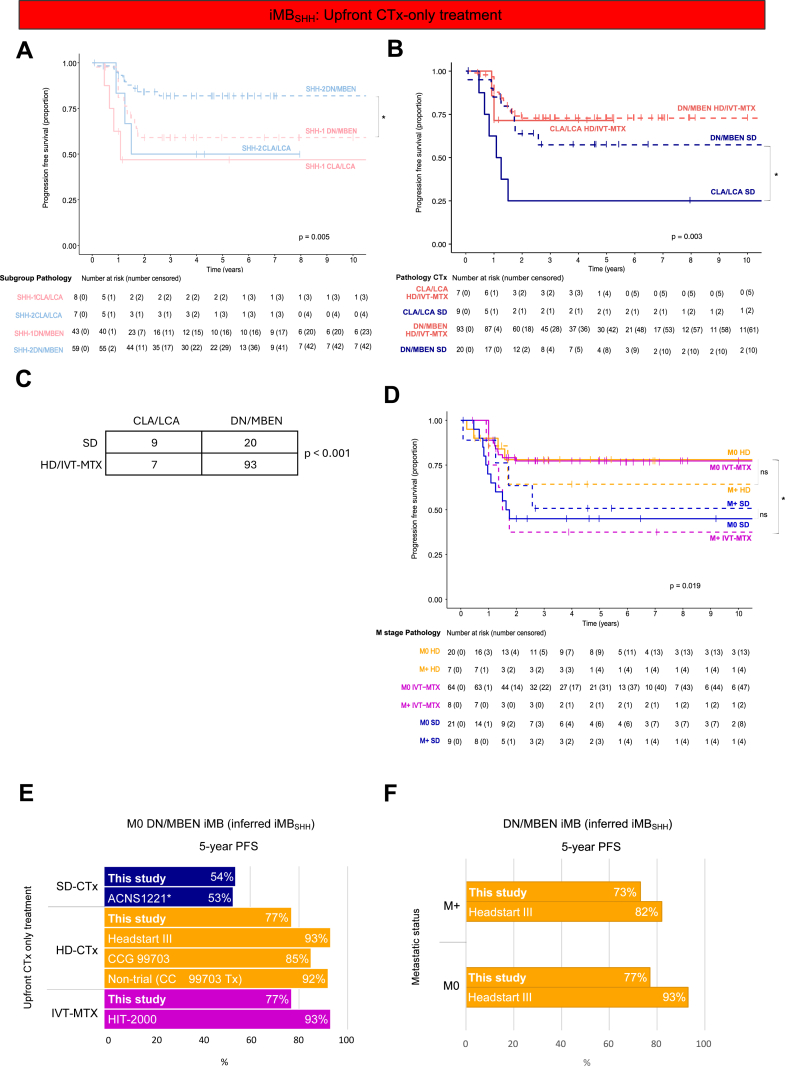
**Exploratory survival analyses of iMB_SHH_ patients receiving upfront CTx-only treatment.** (A) Kaplan–Meier plot of progression-free survival according to SHH molecular subgroup and histological subtypes. At-risk table (numbers censored in parentheses) and p-value for log-rank test are shown. ∗Indicates p-value < 0.05. (B) Kaplan–Meier plot of progression-free survival according to histological subtype and upfront CTx type. At-risk table (numbers censored in parentheses) and p-value for log-rank test are shown. ∗Indicates p-value < 0.05. (C) Relationship between histological subtype and upfront CTx type. p-value for chi-squared test is shown. (D) Kaplan–Meier plot of progression-free survival according to metastatic status and upfront CTx type. At-risk table (numbers censored in parentheses) and p-value for log-rank test are shown. ∗Indicates p-value < 0.05. (E) Mean 5-year progression-free survival proportion of previous M0 DN/MBEN iMB_SHH_ studies according to upfront CTx type. Figure includes previous iMB studies for which PFS information could be extracted for comparable patient cohorts. ∗ACNS1221 study reported 2-year PFS. (F) Mean 5-year progression-free survival proportion of previous DN/MBEN iMB_SHH_ studies according to metastatic status. Figure includes previous iMB studies for which PFS information could be extracted for comparable patient cohorts. CTx = Chemotherapy. CLA = Classic histology. LCA = Large-cell/anaplastic histology. MBEN = Medulloblastoma with extensive nodularity. DN = Desmoplastic nodular. SD = Standard-dose (blue). HD = High-dose (orange). IVT-MTX = Intraventricular methotrexate (pink). M+ = Metastatic disease. M0 = non-metastatic disease. PFS = progression-free survival.

We then undertook targeted exploratory analyses of specific factors within the CTX-only cohort, and their relationships to outcomes. Considering all therapy groups, PFS within the DN/MBEN iMB_SHH_ tumour group was significantly better for SHH-2 than for SHH-1 (5-year PFS; SHH-2 82%, 95% CI 72–93%, SHH-1 59%, 95% CI 46–76%) (Fig. 5A), while PFS and OS of SHH-1 (5-year PFS 47%, 95% CI 22–100%) and SHH-2 (5-year PFS 50%, 95% CI 23–100%) were equivalently poor in non-DN/MBEN (CLA/LCA) tumours (Fig. 1A, Supplementary Fig. S7A). Evaluating the association between pathology group and outcomes within the CTx regimens, DN/MBEN iMB_SHH_ had equivalently good PFS and OS across different CTx types, whilst non-DN/MBEN survival was CTx dependent (Fig. 5B, Supplementary Fig. S7B). PFS and OS for standard-dose CLA/LCA tumours was very poor (5-year OS; standard-dose CLA/LCA 26%, 95% CI 8–85%); an association between CLA/LCA tumours and standard-dose in our cohort was noted (Fig. 5C). Whilst there were limited numbers of high-dose/IVT-MTX CLA/LCA, the survival of these treatment groups appeared to be favourable (Supplementary Fig. S7B). Regarding disseminated disease, IVT-MTX treated metastatic patients did significantly worse compared to those with non-metastatic disease (5-year PFS, M + IVT-MTX 35%, 95% CI 15–92%; M0 IVT-MTX 77%, 95% CI 68–89%, p = 0.013). However, standard-dose and high-dose-CTx treated metastatic patients did not have significantly worse PFS than non-metastatic (Fig. 5D, Supplementary Fig. S7C).

**Fig. 6:**
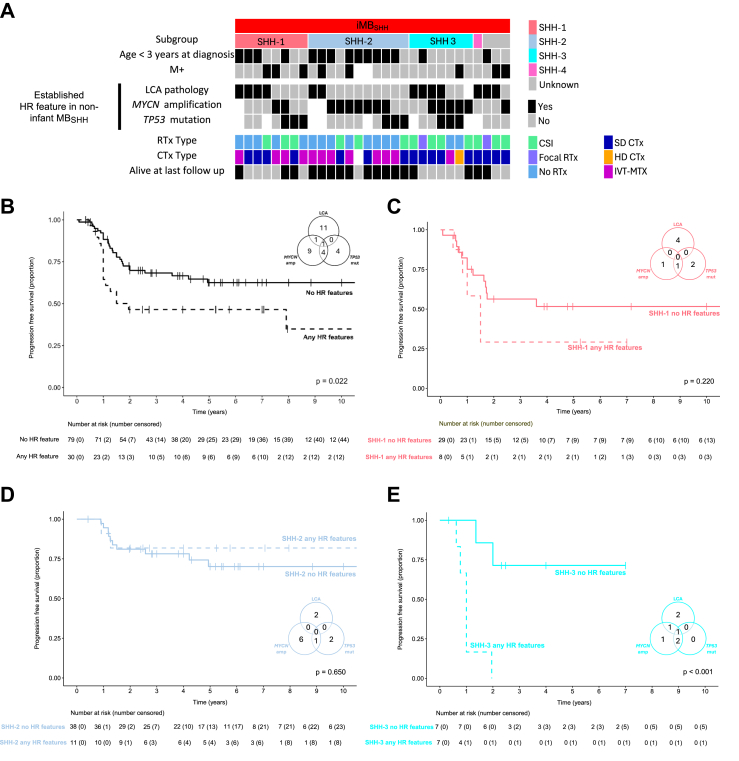
**Nature and clinical behaviour of established SHH group high-risk features in iMB_SHH_ patients.** (A) Oncoplot summarising established SHH group high-risk features in non-infant MB_SHH_. Dark grey bar = positive for feature. Light grey bar = negative for feature. Unfilled = data unavailable. (B) Kaplan–Meier plot of progression-free survival for patients with any high-risk feature versus no high-risk features in iMB_SHH_. At-risk table (numbers censored in parentheses) and p-value for log-rank test are shown. (C) Kaplan–Meier plot of progression-free survival for any high-risk feature versus no high-risk features in iMB SHH-1 group. At-risk table (numbers censored in parentheses) and p-value for log-rank test are shown. Venn diagram shows relationship between individual high-risk features. (D) Kaplan–Meier plot of progression-free survival for any high-risk features versus no high-risk features in iMB SHH-2 group. At-risk table (numbers censored in parentheses) and p-value for log-rank test are shown. Venn diagram shows relationship between individual high-risk features. (E) Kaplan–Meier plot of progression-free survival for any high-risk features versus no high-risk features in iMB SHH-3. At-risk table (numbers censored in parentheses) and p-value for log-rank test are shown. Venn diagram shows relationship between individual high-risk features. M+ = Metastatic disease. HR = High-risk. LCA = Large-cell/anaplastic histology. RTx = Radiotherapy. CTx = Chemotherapy. CSI = Craniospinal irradiation. SD = Standard-dose. HD = High-dose. IVT-MTX = Intraventricular methotrexate.

*TP53* mutations were a feature of all iMB_SHH_ subgroups (Fig. 6). *TP53*-mutated SHH-3 tumours showed the canonical constellation of *TP53*-associated features (*MYCN* amplification, LCA histology) previously recognised as high-risk in the non-infant disease^24^^,^^27^; all of these patients died (3/3; Fig. 6E). All three features were also observed in SHH-1 and SHH-2 but, in contrast, were not associated with each other or with worse survival outcomes (Fig. 6, Supplementary Fig. S8).

In summary, non-metastatic iMB_SHH_ patients in our cohort, treated with upfront high-dose or IVT-MTX regimens, have equivalent favourable 5-year PFS (>75%) and OS (>85%) outcomes (Fig. 5D, Supplementary Fig. S7C), independent of histology and the status of other putative biomarkers (i.e., WHO subgroup, *TP5*3/*MYCN* status). To validate these findings, we integrated and re-evaluated results from previous trials in DN/MBEN iMB, in the context of our findings (Fig. 5E and F; Supplementary Table S1). PFS outcomes observed in our study for different upfront CTx-only regimens, were generalisable to other cohorts; phase-2 trials of high-dose^6^^,^^7^^,^^10^ or IVT-MTX^4^ in non-metastatic cohorts had favourable PFS (5-year PFS; >79%) compared to standard-dose-CTx trials (2-year PFS; 53%).^9^ Outcomes for IVT-MTX in our study (5-years PFS; 73%) differed from the very favourable survival reported for the HIT-2000 trial (5-year PFS; 93%),^4^ but were equivalent to the non-trial validation cohort reported by Mynarek et al.,^4^ suggesting inferior outcomes with this regimen outside the trial setting. We further note the results published to date for the HIT-2000 trial have not included metastatic iMB_SHH_, shown to fare worse with IVT-MTX in our cohort.

## Discussion

The absence of large-scale or randomised trials in iMB has hindered the evidence-based development of optimal therapies which balance maximal cure with minimal late-effects. Our strategy to assemble a comprehensive real-world experience in iMB_SHH_, together with re-examination of historical trials data and careful cross-interpretation, has allowed us to directly compare different therapy concepts from recent smaller phase 2 trials,^4^^,^^6^^,^^7^^,^^9^^,^^10^ and resolve the biomarker and therapy-dependent nature of iMB_SHH_ survival outcomes.

Radiation-sparing chemotherapy-only approaches were the most common foundation of upfront iMB_SHH_ therapy in our cohort, strongly associated with patients under 3 years of age at diagnosis, and aimed at the avoidance or delay of CSI. In this treatment group, PFS was strongly associated with CTx type; outcomes were significantly improved using intensified chemotherapy. Upfront CSI produced no further upfront progression-free survival (PFS) benefit compared to high-dose and IVT-MTX-based regimens, underlining the rationale for these approaches to minimise therapy-associated late-effects.^1^^,^^4^, ^5^, ^6^, ^7^^,^^10^

Prognostic factors for radiation-sparing iMB_SHH_ treatments were therapy-dependent. Intensified high-dose or IVT-MTX chemotherapy was associated with better PFS in patients with SHH-1 subgroup tumours while, in contrast, SHH-2 disease was more chemo-sensitive and had good outcomes independent of CTx type. Similarly, in our exploratory analyses, non-DN/MBEN disease had significantly worse PFS using standard-dose-CTx, but was equivalent to DN/MBEN when using intensified therapies; metastatic disease was only associated with a significantly worse PFS in patients treated with IVT-MTX. Recent findings from the ACNS0334 trial, which described 11/11 iMB_SHH_ survivors (6 with metastatic disease) following high-dose chemotherapy, add further support to our findings.^28^

The prognostic significance of high-risk factors previously reported for non-infant MB_SHH_ (*TP53* mutation, *MYCN* amplification and LCA pathology),^15^^,^^27^ was subgroup-dependent. Although we identified these in all iMB_SHH_ subgroups, their association with each other, and with worse outcomes, was restricted to the older SHH-3 - which was rare in the infant setting.

Together, our findings support the inclusion of iMB_SHH_ patients (i.e., all SHH-1 and SHH-2 subgroup members, and all pathology variants) in a single treatment group with favourable-risk when treated with intensified chemotherapy-only approaches. No evidence was found to support non-DN/MBEN pathology, subgroup, or *TP5*3/MYCN/LCA status as significant risk-factors in SHH-1/SHH-2 tumours using these treatments; these cases should be included, but careful monitoring of their outcomes is recommended in future studies. In contrast, metastatic disease should not presently be considered favourable-risk due to the relatively limited numbers of cases examined, and inferior outcomes observed using IVT-MTX – further experience is required, ideally through their inclusion in clinical trials for high-risk iMB (e.g., SIOP-PNOC-TRIUMPH). Current recommendations from SIOP-Europe and the European Reference Network are thus to use high-dose therapies for iMB_SHH_ patients with metastatic disease.^29^

These results also have potential consequences in resource limited settings where biological information is increasingly available to support therapy stratification. The ability to use more easily deliverable standard-dose chemotherapy confidently in those patients that do not need more intensive therapy (i.e., SHH-2 patients), could enable concentration of limited resources to deliver more intensive therapies only to those who require it.

We recognise certain limitations in our study. Our investigations encompassed retrospective patient cohorts treated using different protocols; unmeasured confounding and other biases of such observational research are thus a principal limitation in contrast to randomised trials. However, we note findings for the therapy groups we defined validated across our cohort and our re-evaluation of recent trials, where possible. The inter-relationship between age and treatment factors introduces potential bias in their comparison, and dictated the focus on treatment-dependent analyses reported here. While our retrospective analysis did allow an indicative comparison of chemotherapy-only treatments with CSI-based therapies, this must be interpreted with due caution. Whether patients had second look surgery or only one surgical resection was not recorded although the presence of residual disease at the point of diagnosis was included in the analysis. Those receiving radiotherapy at the end of chemotherapy due either to residual disease or physician discretion were known; patients receiving radiotherapy prior to relapse were excluded from the ‘chemotherapy-only’ group. Furthermore, information on pattern of relapse and treatments received at recurrence was only available for a small proportion of our cohort. In some countries, relapsed iMB treated with IVT-MTX upfront would not typically receive CSI as rescue treatment due to the toxicity and neurocognitive deficit associated with CSI after IVT-MTX.^30^ Finally, as race and ethnicity data were not collected across contributing centres, we were unable to assess representativeness with respect to these sociocultural factors.

Our cohort overlaps in part with the HIT-2000 non-trial validation cohort^4^^,^^8^ we refer to, however, we note these patients made up only a proportion (n = 32/72; 44%) of our multi-centre IVT-MTX treated cohort. Furthermore, inferior outcomes with this regimen outside the trial setting have been previously reported,^31^ raising adherence to protocol outside of the trial setting as a potential issue. The importance of germline mutations, particularly *TP53* status, could not be assessed. Finally, we note that currently many lower- and middle-income countries do not have access to contemporary molecular diagnostics used in higher-income countries; in addition, intraventricular therapy and high dose therapy are challenging to deliver. Together these limit the immediate application of the findings of this study in these settings.

Following removal of patients with metastatic disease, the favourable-risk iMB_SHH_ M0 group treated with high-dose or IVT-MTX had a 5-year PFS of 77% in our cohort, and validated in historical trials cohorts (77–93%; Fig. 5D–F). Critically, this upfront cure negates the need for any rescue therapies (including CSI) in the overwhelming majority, and their associated toxicities. With favourable outcomes established using both intensified therapeutic strategies, our study supports a sea change in the emphasis of iMB_SHH_ clinical trials. Upcoming trials, such as SIOP-CONNECT-COGNITO-MB and SJiMB21 (NCT05535166), will now focus on randomised comparisons of quality-of-life and neurocognitive endpoints associated with different intensified therapies, to identify kindest curative strategies.

## Contributors

Conception and design: SR, DH, ECS, SB and SCC. Collection and assembly of data: SR, DH, MG, JC, SC, MGA, IMG, SLAP, FB, CD, JMP, FRB, VB, MM, KI, YK, VR, AG, AK, SMP, MM, SRu, SB, SCC. SR, DH and SCC accessed and verified the underlying data. All authors have access to all the data. Data analysis and interpretation: SR, DH, MG, ERB, DT, ECS, SB and SCC. Manuscript writing: All authors. Final approval of manuscript: All authors. Accountable for all aspects of the work: All authors.

## Data sharing statement

Raw and processed DNA methylation microarray data and associated patient metadata have been deposited in the NCBI Gene Expression Omnibus (GEO) with accession number GSE317378. This submission also contains data and metadata from our study on non-WNT/non-SHH infant medulloblastoma.^18^

## Declaration of interests

SCC reports funding support for the study from Cancer Research UK, Children with Cancer UK, Children’s Cancer North, Star for Harris, JGW Patterson Foundation, Little Hero and Blue Skye Thinking. AG reports participation on a Data Safety Monitoring Board for Day One Therapeutics. SR reports support for the present manuscript from the German Children’s Cancer Foundation; grants from BMBF; consulting fees from Fennec Pharma and Norgine GmbH; honoraria from Norgine; and meeting attendance support from Norgine. All other authors declare no interests.

## Appendix A Supplementary data

Supplementary Figs. S1–S8 **Supplementary Figure 1:** (A) Kaplan-Meier plot of progression-free survival for patients treated with upfront CSI + chemotherapy in our study cohort (This study) compared to a combined cohort of previous studies of iMB_SHH_ sourced from Smith et al. Neuro-oncology, 2025 (ACNS0331, ACNS0332, SJMB03). **Supplementary Figure 2:** (A) Frequency of CTx regimens used in upfront CTx-only treatments for iMB_SHH_. (B) Number of patients in upfront treatment groups greater or less than 3 years of age at diagnosis, shown for individual contributing countries. (C) Kaplan-Meier plot of overall survival for iMB_SHH_ upfront treatment groups: CSI +CTx, Focal +CTx, HD-CTx only, IVT-MTX only and SD-CTx only. At-risk tables (number censored in parentheses) and p-values from log-rank tests are shown. ∗ Indicates p-value <0.05. (D) Kaplan-Meier plot of post-relapse survival for iMB_SHH_ upfront treatment groups: CSI +CTx, Focal +CTx, HD-CTx only, IVT-MTX only and SD-CTx only. At-risk tables (number censored in parentheses) and p-values from log-rank tests are shown. CTx = Chemotherapy. RTx = Radiotherapy. CSI = Craniospinal irradiation. SD = Standard-dose. HD = High-dose. IVT-MTX = Intraventricular methotrexate. **Supplementary Figure 3:** (A) Kaplan-Meier plot of progression-free survival (PFS) and overall survival (OS) for iMB_SHH_ patients treated with upfront CSI and CTx. (B) Kaplan-Meier plot of progression-free survival (PFS) and overall survival (OS) for iMB_SHH_ patients treated with upfront Focal RTx and CTx. (C) Kaplan-Meier plot of progression-free survival (PFS) and overall survival (OS) for iMB_SHH_ patients treated with upfront HD-CTx only. (D) Kaplan-Meier plot of progression-free survival (PFS) and overall survival (OS) for iMB__SHH__ patients treated with upfront IVT-MTX only. (E) Kaplan-Meier plot of progression-free survival (PFS) and overall survival (OS) for iMB_SHH_ patients treated with upfront SD-CTx only. At-risk tables are shown (number censored in parentheses). CTx = Chemotherapy. RTx = Radiotherapy. CSI = Craniospinal irradiation. SD = Standard-dose. HD = High-dose. IVT-MTX = Intraventricular methotrexate. **Supplementary Figure 4:** (A) Oncoplot representing clinico-pathological and molecular disease features of our cohort. Residuals and p-values from chi-squared tests are shown (darker shades indicate stronger relationships). CTx = Chemotherapy. RTx = Radiotherapy. CSI = Craniospinal irradiation. SD = Standard-dose. HD = High-dose. IVT-MTX = Intraventricular methotrexate. GTR = Gross-total resection. CLA = Classic histology. LCA = Large-cell/anaplastic histology. MBEN = Medulloblastoma with extensive nodularity. DN = Desmoplastic nodular. M+ = Metastatic disease. **Supplementary Figure 5:** (A) Kaplan-Meier plot of overall survival in upfront CSI + CTx treated iMB_SHH_ according to DN/MBEN versus CLA/LCA pathological subtypes. At-risk tables (numbers censored in parentheses) and p-values from log-rank tests are shown. (B) Kaplan-Meier plot of overall survival in upfront CSI + CTx treated iMB_SHH_ according to SHH molecular subgroup. At-risk tables (numbers censored in parentheses) and p-values from log-rank tests are shown. (C) Kaplan-Meier plot of overall survival in in upfront CSI + CTx treated iMB_SHH_ according to *MYCN* amplification status. At-risk tables (numbers censored in parentheses) and p-values from log-rank tests are shown. CTx = Chemotherapy. CSI = Craniospinal irradiation. CLA = Classic histology. LCA = Large-cell/anaplastic histology. MBEN = Medulloblastoma with extensive nodularity. DN = Desmoplastic nodular. **Supplementary Figure 6:** (A) Kaplan-Meier plot of overall survival for upfront CTx-only treated iMB SHH-1 according to CTx type. At-risk table (numbers censored in parentheses) and p-value for log-rank test are shown. (B) Kaplan-Meier plot of overall survival for upfront CTx-only treated iMB SHH-2 according to CTx type. At-risk table (numbers censored in parentheses) and p-value for log-rank test are shown. ∗ Indicates p-value <0.05. CTx = Chemotherapy. SD = Standard-dose. HD = High-dose. IVT-MTX = Intraventricular methotrexate. **Supplementary Figure 7:** (A) Kaplan-Meier plot of overall survival for upfront CTx-only treated iMB_SHH_ according to SHH molecular subgroup and histological subtypes. At-risk table (numbers censored in parentheses) and p-value for log-rank test are shown. ∗Indicates p-value < 0.05. ∗∗p-value 0.01–0.05. ∗∗∗p-value < 0.001. (B) Kaplan-Meier plot of overall survival for upfront CTx-only treated iMB_SHH_ according to histological subtype and upfront CTx type. At-risk table (numbers censored in parentheses) and p-value for log-rank test are shown. ∗ Indicates p-value <0.05. ∗∗∗p-value < 0.001. (C) Kaplan-Meier plot of overall survival for upfront CTx-only treated iMB_SHH_ according to metastatic status and upfront CTx type. At-risk table (numbers censored in parentheses) and p-value for log-rank test are shown. CTx = Chemotherapy. SD = Standard-dose. HD = High-dose. IVT-MTX = Intraventricular methotrexate. CLA = Classic histology. LCA = Large-cell/anaplastic histology. MBEN = Medulloblastoma with extensive nodularity. DN = Desmoplastic nodular. M+ = Metastatic disease. M0 = non-metastatic disease. **Supplementary Figure 8:** (A) Kaplan-Meier plot of progression-free survival for LCA pathology in iMB_SHH_. At-risk table (numbers censored in parentheses) and p-value for log-rank test are shown. (B) Kaplan-Meier plot of progression-free survival for *MYCN* amplification status in iMB_SHH_. At-risk table (numbers censored in parentheses) and p-value for log-rank test are shown. (C) Kaplan-Meier plot of progression-free survival for *TP53* mutation status in iMB_SHH_. At-risk table (numbers censored in parentheses) and p-value for log-rank test are shown. (D) Summary of demographic/molecular features and survival for *TP53* mutant iMB_SHH_ cases. Dark grey bar = positive for feature. Light grey bar = negative for feature. Unfilled = data unavailable. LCA = Large-cell/anaplastic histology. CLA = Classic histology. MBEN = Medulloblastoma with extensive nodularity. DN = Desmoplastic nodular. CTx = Chemotherapy. SD = Standard-dose. HD = High-dose. IVT-MTX = Intraventricular methotrexate.
