# Supplementary material for: Therapy and biomarker dependent progression-free survival in infant sonic hedgehog medulloblastoma: a multi-national retrospective cohort study
Source: eClinicalMedicine. 2026 May 18;96:103913. doi: 10.1016/j.eclinm.2026.103913 (PMC13316349; doi:10.1016/j.eclinm.2026.103913)

Supplementary figure 1

A

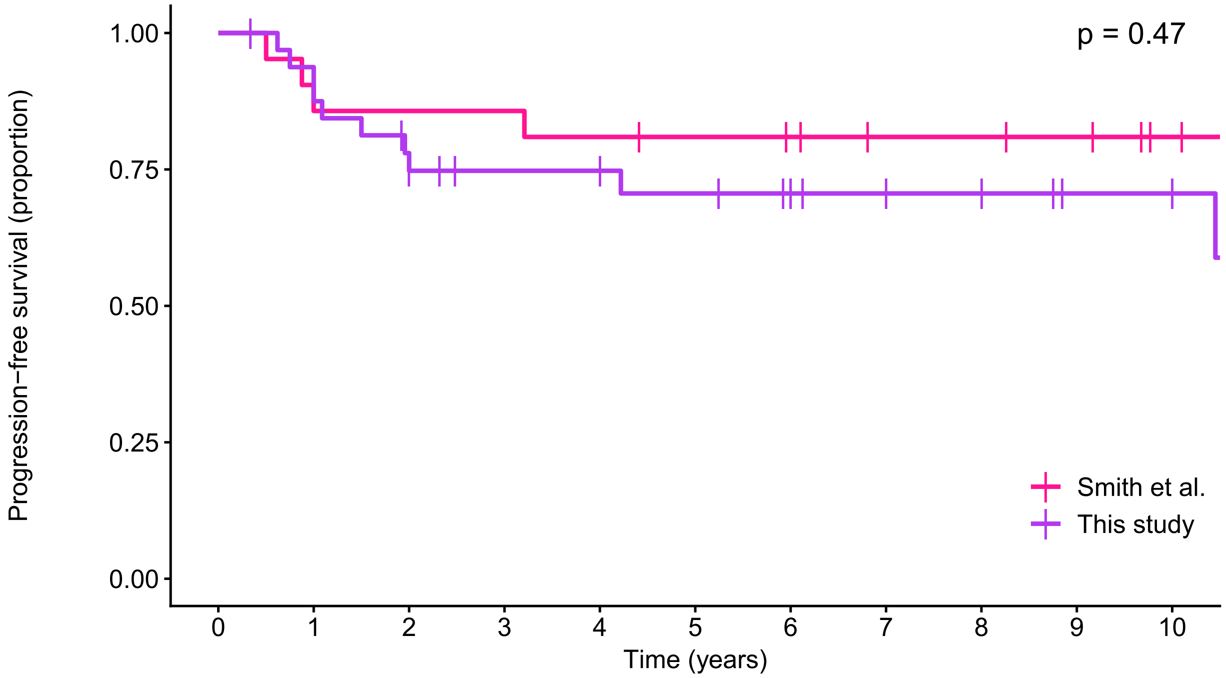

Number at risk (number censored)

Smith et al.	21 (0)	18 (0)	18 (0)	18 (0)	17 (0)	16 (1)	15 (2)	13 (4)	13 (4)	12 (5)	8 (9)
This study	33 (0)	30 (1)	24 (4)	19 (6)	19 (7)	17 (7)	15 (10)	13 (13)	11 (15)	7 (17)	7 (18)

Supplementary figure 2

A

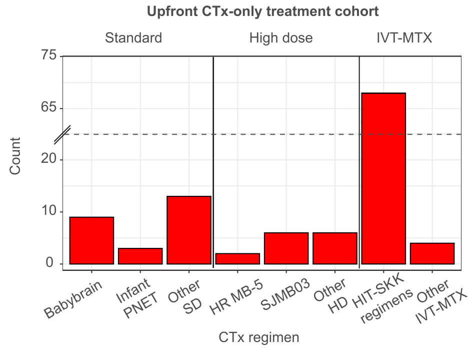

B

Contributing cohort	CSI + CTx		Focal RTx + CTx		CTx only	
	≥ 3 years	< 3 years	≥ 3 years	< 3 years	≥ 3 years	< 3 years
Canada	9 (26%)	8% (42%)	1 (33%)	5 (22%)	1 (6%)	18 (16%)
France	0 (0%)	0 (0%)	0 (0%)	0 (0%)	6 (35%)	20 (17%)
Italy	0 (0%)	1 (5%)	0 (0%)	1 (4%)	0 (0%)	2 (2%)
Japan	3 (9%)	3 (16%)	0 (0%)	0 (0%)	1 (6%)	13 (11%)
The Netherlands	1 (3%)	0 (0%)	0 (0%)	0 (0%)	3 (18%)	7 (6%)
Spain	2 (6%)	2 (11%)	0 (0%)	1 (4%)	0 (0%)	1 (1%)
UK	9 (26%)	4 (21%)	2 (67%)	16 (70%)	0 (0%)	23 (20%)
USA/Germany	11 (31%)	1 (5%)	0 (0%)	0 (0%)	6 (35%)	32 (28%)
Total	35 (100%)	19 (100%)	3 (100%)	23 (100%)	17 (100%)	116 (100%)

C

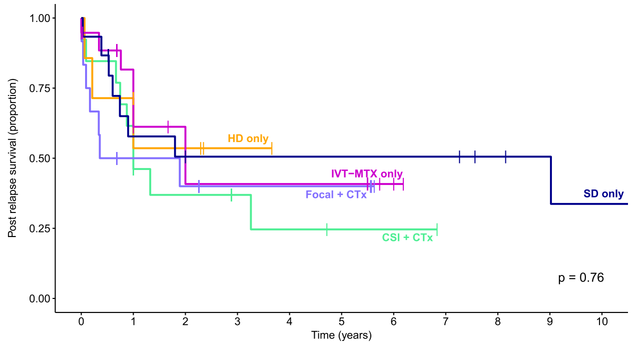

Number at risk (number censored)

	0	1	2	3	4	5	6	7	8	9	10
CSI +CTx	13 (0)	8 (1)	4 (1)	3 (2)	2 (2)	1 (3)	1 (3)	0 (4)	0 (4)	0 (4)	0 (4)
Focal + CTx	12 (0)	5 (1)	4 (1)	3 (2)	3 (2)	3 (2)	0 (5)	0 (5)	0 (5)	0 (5)	0 (5)
HD only	7 (0)	4 (1)	3 (1)	1 (3)	0 (4)	0 (4)	0 (4)	0 (4)	0 (4)	0 (4)	0 (4)
IVT−MTX only	19 (2)	11 (6)	6 (7)	4 (7)	4 (7)	4 (7)	2 (10)	0 (11)	0 (11)	0 (11)	0 (11)
SD only	15 (0)	8 (1)	7 (2)	6 (2)	6 (2)	6 (2)	6 (2)	6 (2)	4 (4)	3 (5)	2 (5)

D

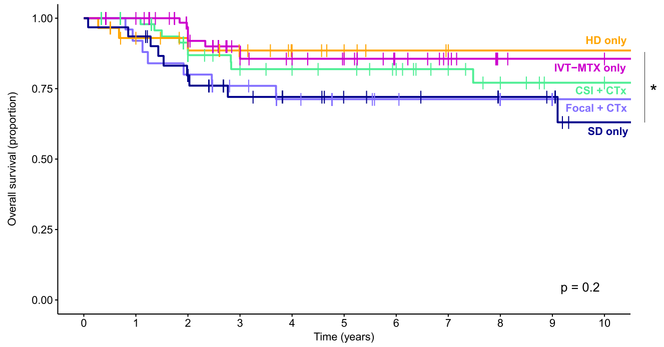

Number at risk (number censored)

	0	1	2	3	4	5	6	7	8	9	10
CSI + CTx	49 (0)	47 (2)	41 (6)	33 (9)	31 (11)	29 (12)	26 (16)	21 (23)	15 (27)	10 (30)	10 (31)
Focal + CTx	25 (0)	23 (0)	20 (0)	17 (2)	14 (4)	12 (6)	10 (8)	9 (9)	9 (10)	8 (11)	7 (11)
HD only	29 (0)	25 (4)	21 (7)	17 (9)	13 (14)	10 (18)	6 (20)	5 (22)	4 (22)	4 (22)	4 (22)
IVT−MTX only	72 (0)	71 (2)	60 (19)	41 (29)	33 (35)	27 (39)	19 (45)	15 (51)	8 (56)	7 (57)	7 (60)
SD only	31 (0)	29 (0)	23 (3)	18 (5)	16 (7)	13 (10)	12 (11)	11 (12)	10 (13)	9 (14)	5 (17)

Supplementary figure 3

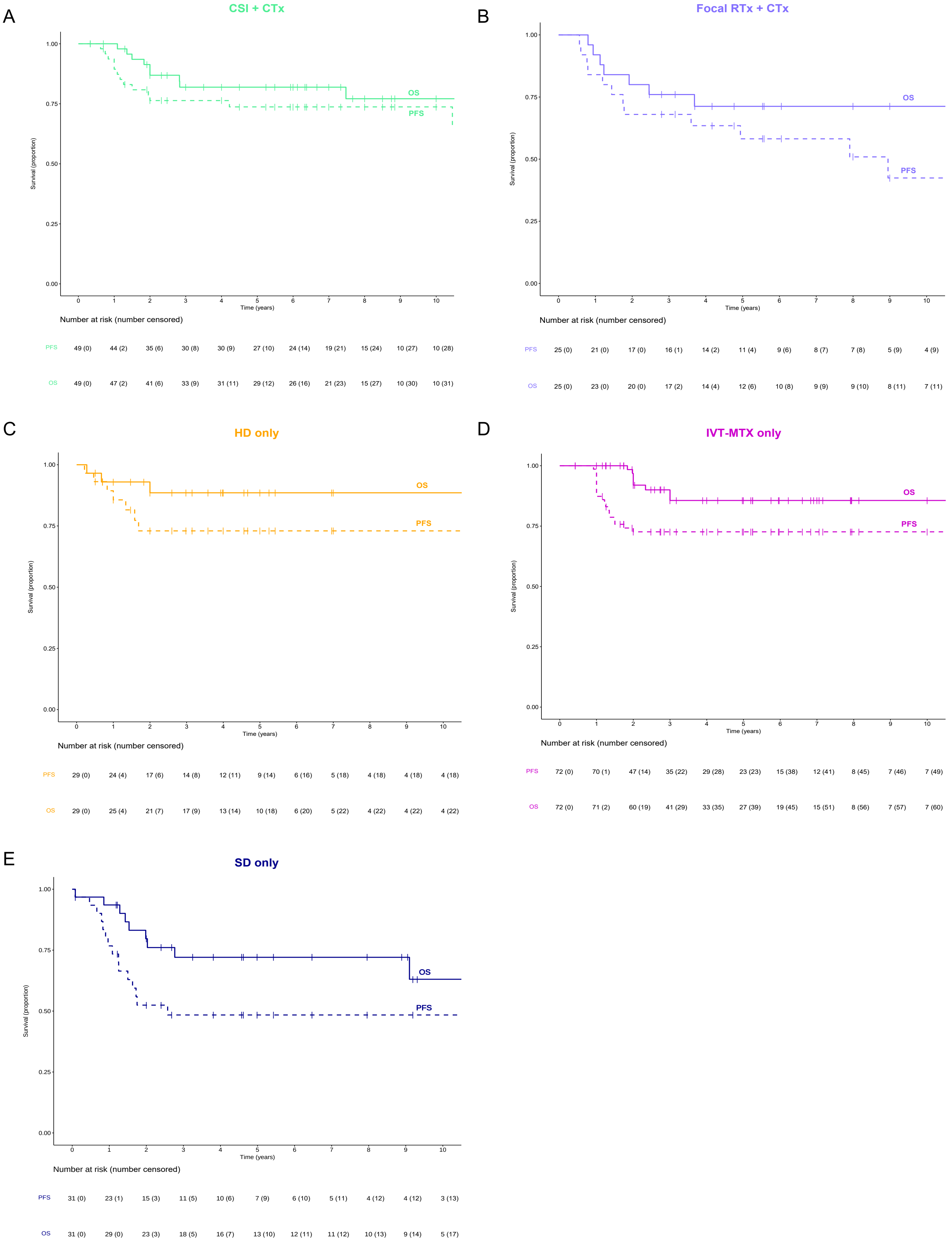

Supplementary figure 4

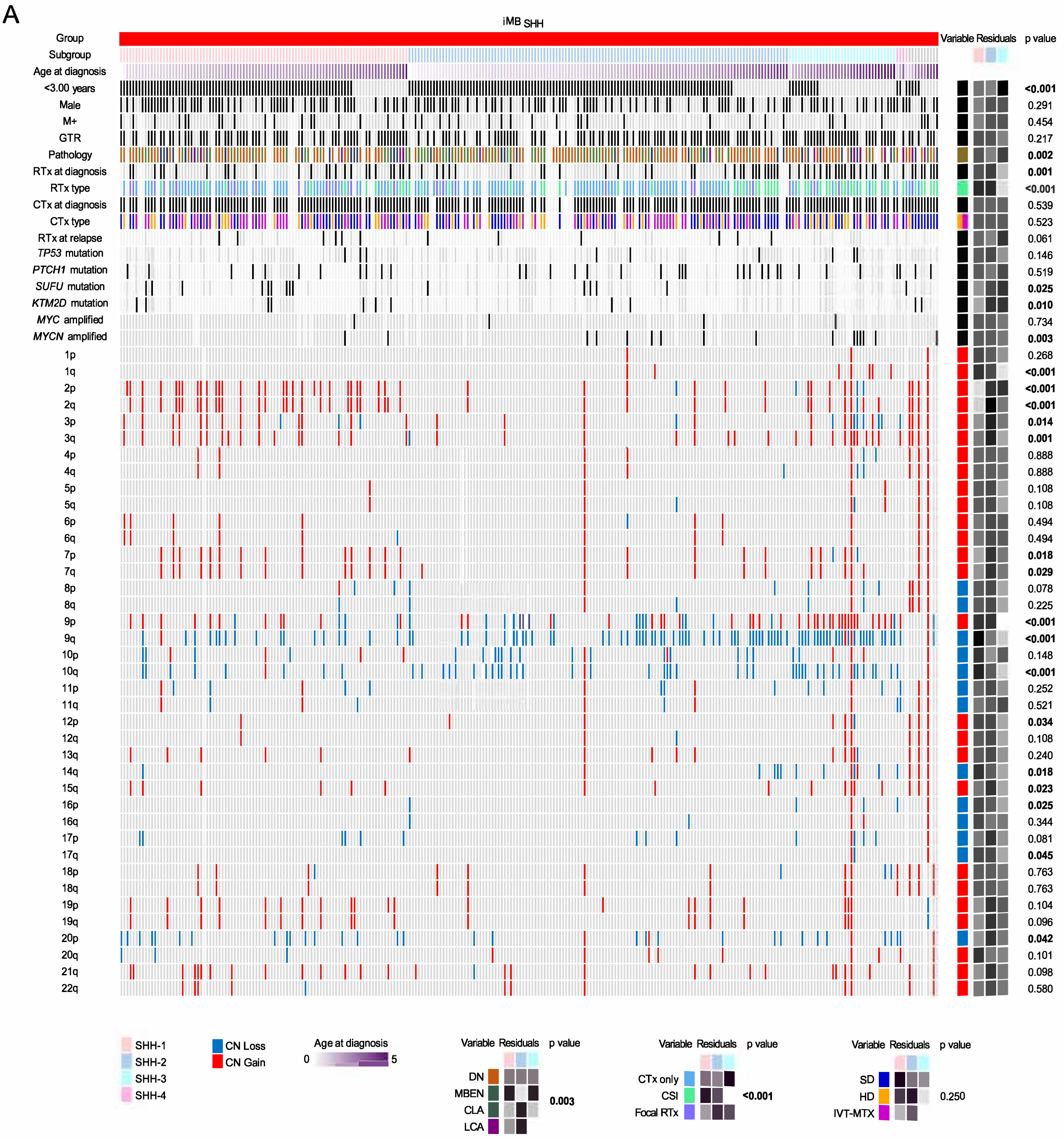

Supplementary figure 5

iMB$_{SHH}$: Upfront CSI and CTx treatment

A

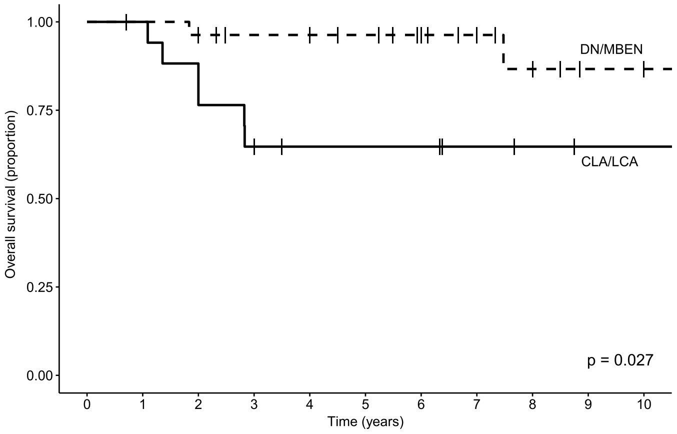

Number at risk (number censored)

	0	1	2	3	4	5	6	7	8	9	10
DN/MBEN	28 (0)	27 (1)	26 (3)	22 (5)	22 (6)	20 (7)	17 (11)	14 (16)	9 (19)	5 (21)	5 (22)
CLA/LCA	17 (0)	17 (0)	15 (0)	11 (1)	9 (2)	9 (2)	9 (2)	7 (4)	6 (5)	5 (6)	5 (6)

B

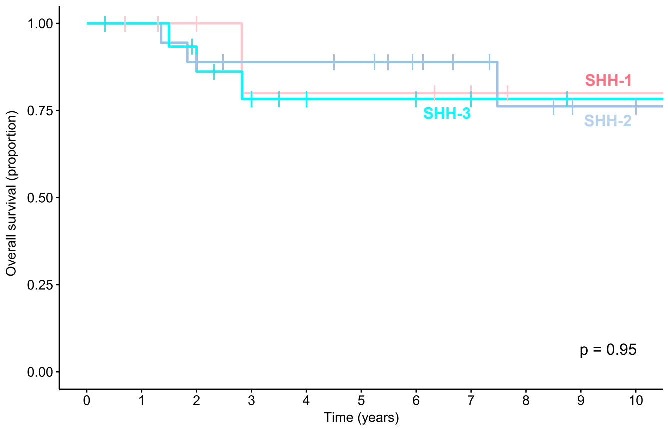

Number at risk (number censored)

	0	1	2	3	4	5	6	7	8	9	10
SHH-1	8 (0)	7 (1)	6 (3)	4 (3)	4 (3)	4 (3)	4 (3)	3 (5)	1 (6)	1 (6)	1 (6)
SHH-2	18 (0)	18 (0)	16 (1)	14 (2)	14 (2)	13 (3)	10 (6)	8 (8)	6 (9)	4 (11)	4 (12)
SHH-3	16 (0)	15 (1)	13 (2)	10 (4)	8 (6)	7 (6)	7 (7)	6 (9)	4 (9)	3 (10)	3 (10)

C

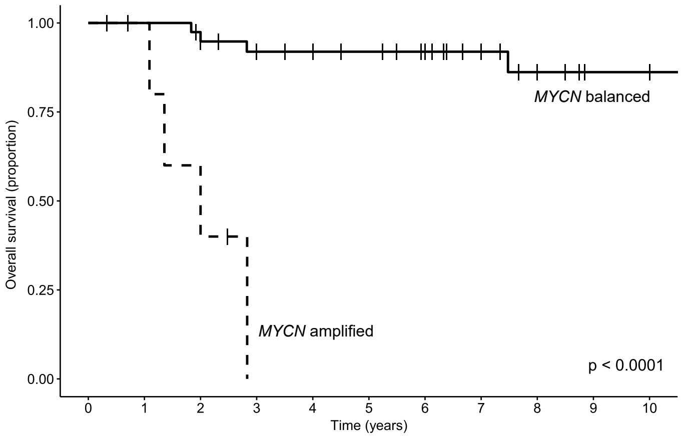

Number at risk (number censored)

	0	1	2	3	4	5	6	7	8	9	10
MYCN amplified	7 (0)	5 (2)	3 (2)	0 (3)	0 (3)	0 (3)	0 (3)	0 (3)	0 (3)	0 (3)	0 (3)
MYCN balanced	39 (0)	39 (0)	37 (3)	32 (5)	30 (7)	28 (8)	25 (12)	20 (19)	14 (23)	9 (26)	9 (27)

Supplementary figure 6

iMB$_{SHH}$: Upfront CTx-only treatment

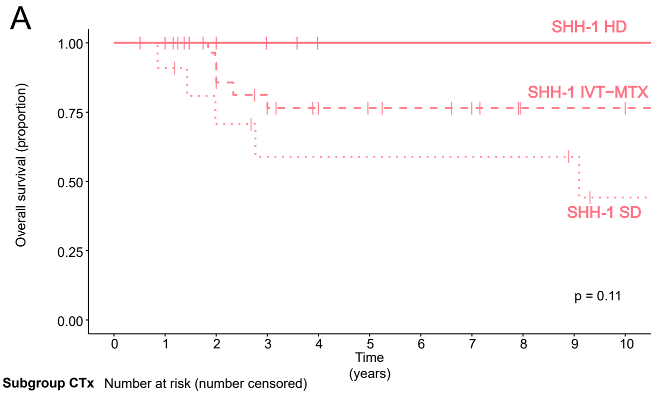

Subgroup CTx	Number at risk (number censored)										
SHH-1 HD	8 (0)	7 (2)	5 (4)	3 (5)	1 (7)	1 (7)	1 (7)	1 (7)	1 (7)	1 (7)	1 (7)
SHH-1 IVT−MTX	32 (0)	32 (0)	26 (9)	17 (11)	13 (14)	11 (15)	10 (16)	9 (18)	5 (21)	5 (21)	5 (24)
SHH-1 SD	11 (0)	10 (0)	7 (1)	5 (2)	5 (2)	5 (2)	5 (2)	5 (2)	5 (2)	4 (3)	2 (4)

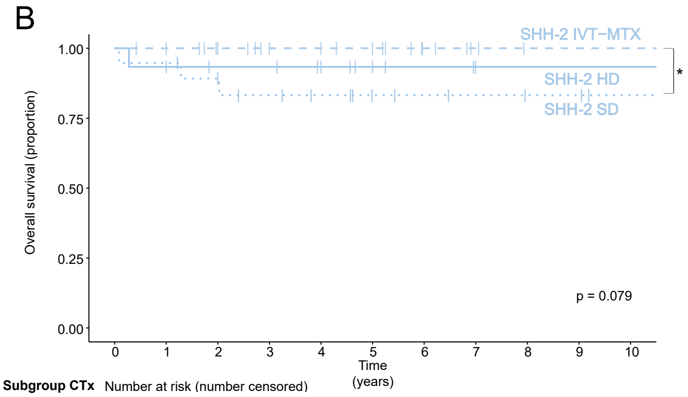

Subgroup CTx	Number at risk (number censored)										
SHH-2 HD	15 (0)	14 (1)	12 (2)	12 (2)	10 (5)	7 (8)	5 (9)	4 (11)	3 (11)	3 (11)	3 (11)
SHH-2 IVT−MTX	33 (0)	32 (2)	28 (9)	21 (15)	18 (18)	14 (21)	7 (26)	4 (29)	2 (31)	2 (31)	2 (31)
SHH-2 SD	19 (0)	18 (0)	16 (2)	13 (3)	11 (5)	8 (8)	7 (9)	6 (10)	5 (11)	5 (11)	3 (13)

Supplementary figure 7

iMB$_{SHH}$: Upfront CTx-only treatment

A

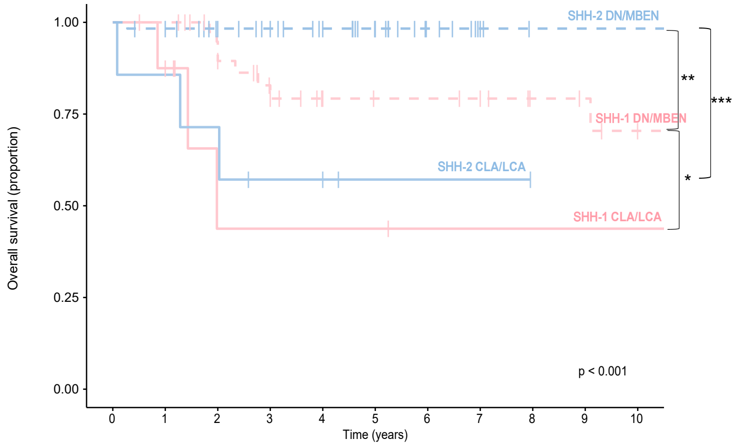

Number at risk (number censored)

	0	1	2	3	4	5	6	7	8	9	10
SHH-1 CLA/LCA	8 (0)	7 (1)	2 (3)	2 (3)	2 (3)	2 (3)	1 (4)	1 (4)	1 (4)	1 (4)	1 (4)
SHH-2 CLA/LCA	7 (0)	6 (0)	5 (0)	3 (1)	3 (2)	1 (3)	1 (3)	1 (3)	0 (4)	0 (4)	0 (4)
SHH-1 DN/MBEN	43 (0)	42 (1)	36 (11)	23 (15)	17 (20)	15 (21)	15 (21)	14 (23)	10 (26)	9 (27)	7 (31)
SHH-2 DN/MBEN	59 (0)	57 (3)	50 (13)	42 (19)	35 (26)	27 (34)	17 (41)	12 (47)	9 (49)	9 (49)	8 (50)

B

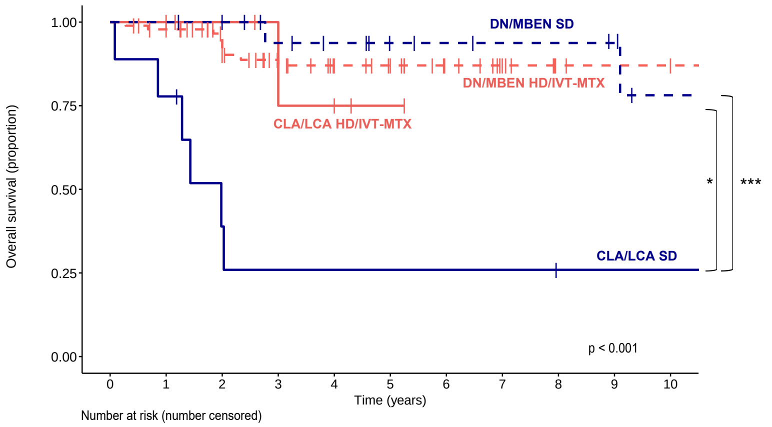

Number at risk (number censored)

	0	1	2	3	4	5	6	7	8	9	10
DN/MBEN HD/IVT-MTX	7 (0)	7 (1)	5 (2)	4 (3)	3 (4)	1 (5)	0 (6)	0 (6)	0 (6)	0 (6)	0 (6)
DN/MBEN SD	9 (0)	7 (0)	3 (1)	2 (1)	2 (1)	2 (1)	2 (1)	2 (1)	1 (2)	1 (2)	1 (2)
CLA/LCA HD/IVT-MTX	93 (0)	88 (5)	75 (24)	53 (35)	42 (45)	35 (52)	25 (58)	20 (66)	12 (71)	11 (72)	11 (75)
CLA/LCA SD	20 (0)	20 (0)	19 (2)	15 (4)	13 (6)	10 (9)	9 (10)	8 (11)	8 (11)	7 (12)	4 (14)

C

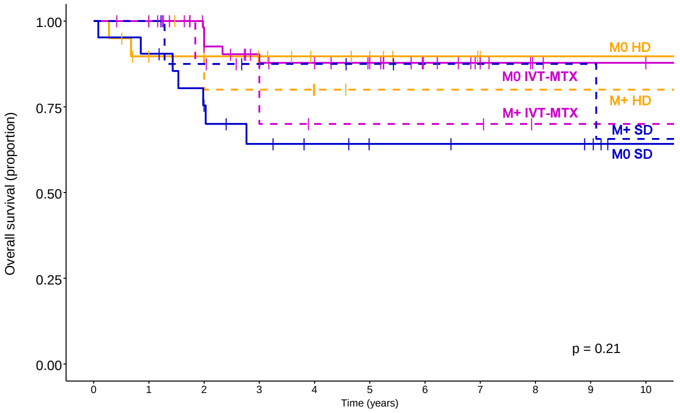

Number at risk (number censored)

	0	1	2	3	4	5	6	7	8	9	10
M0 HD	20 (0)	16 (3)	15 (4)	13 (5)	10 (8)	9 (11)	5 (13)	4 (15)	3 (15)	3 (15)	3 (15)
M+ HD	7 (0)	7 (1)	5 (2)	4 (2)	3 (4)	1 (5)	1 (5)	1 (5)	1 (5)	1 (5)	1 (5)
M0 IVT−MTX	64 (0)	63 (2)	53 (19)	36 (27)	30 (32)	24 (36)	16 (42)	12 (48)	7 (51)	6 (52)	6 (55)
M+ IVT−MTX	8 (0)	8 (0)	7 (0)	5 (2)	3 (3)	3 (3)	3 (3)	3 (3)	1 (5)	1 (5)	1 (5)
M0 SD	21 (0)	19 (0)	15 (2)	11 (3)	9 (5)	7 (7)	7 (7)	6 (8)	6 (8)	5 (9)	2 (12)
M+ SD	9 (0)	9 (0)	7 (1)	6 (2)	6 (2)	5 (3)	4 (4)	4 (4)	4 (4)	4 (4)	3 (4)

Supplementary figure 8

A

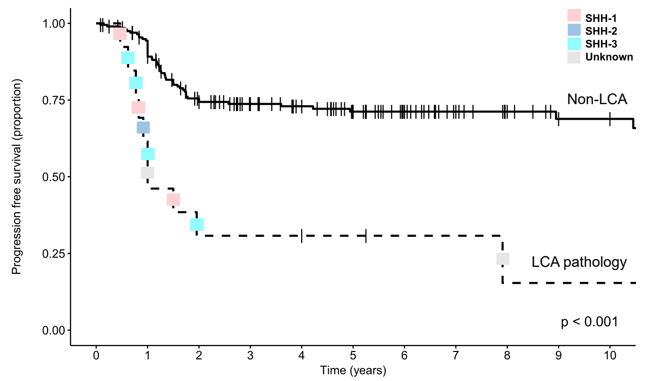

Number at risk (number censored)

Non-LCA	201 (0)	181 (12)	133 (33)	106 (50)	93 (63)	76 (76)	61 (90)	48 (106)	37 (115)	29 (120)	28 (125)
LCA pathology	13 (0)	8 (0)	4 (0)	4 (0)	4 (1)	3 (1)	2 (2)	2 (2)	1 (2)	1 (2)	1(2)

B

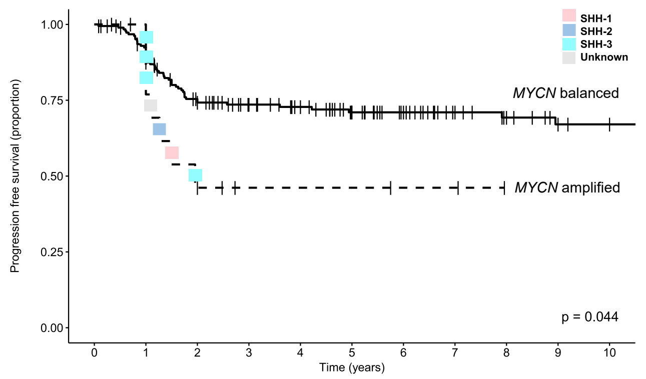

Number at risk (number censored)

MYCN balanced	189 (0)	168 (10)	128 (27)	104 (42)	92 (54)	74 (67)	61 (80)	48 (96)	38 (108)	30 (108)	28 (114)
MYCN amplified	15 (0)	13 (2)	6 (3)	3 (5)	3 (5)	3 (5)	2 (6)	2 (6)	0 (8)	0 (8)	0 (8)

C

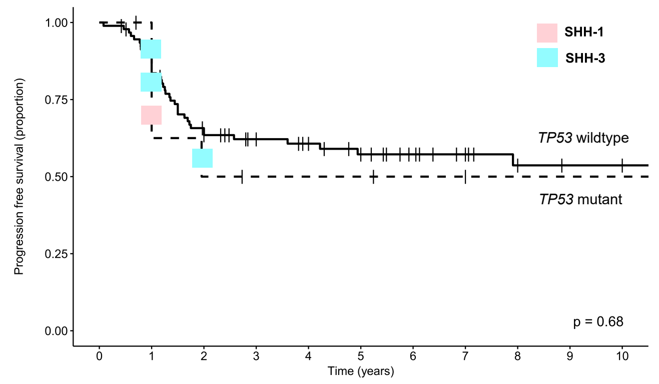

Number at risk (number censored)

TP53 wildtype	93 (0)	81 (2)	58 (8)	45 (16)	40 (22)	32 (26)	25 (31)	21 (38)	15 (42)	12 (43)	12 (47)
TP53 mutant	9 (0)	8 (1)	4 (1)	3 (2)	3 (2)	3 (2)	2 (3)	2 (4)	1 (4)	1 (4)	1 (4)

D

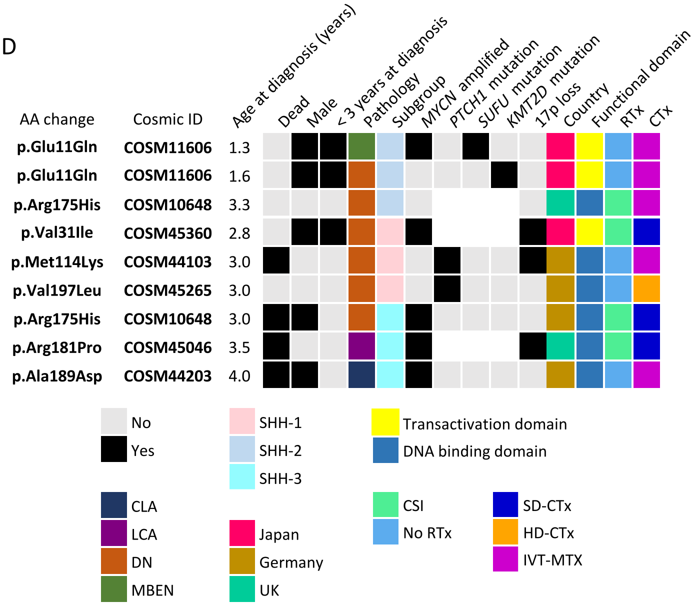

Supplement: Supplementary Figs. S1–S8 — Supplementary Figure 1: (A) Kaplan-Meier plot of progression-free survival for patients treated with upfront CSI + chemotherapy in our study cohort (This study) compared to a combined cohort of previous studies of iMBSHH sourced from Smith et al. Neuro-oncology, 2025 (ACNS0331, ACNS0332, SJMB03). Supplementary Figure 2: (A) Frequency of CTx regimens used in upfront CTx-only treatments for iMBSHH. (B) Number of patients in upfront treatment groups greater or less than 3 years of age at diagnosis, shown for individual contributing countries. (C) Kaplan-Meier plot of overall survival for iMBSHH upfront treatment groups: CSI +CTx, Focal +CTx, HD-CTx only, IVT-MTX only and SD-CTx only. At-risk tables (number censored in parentheses) and p-values from log-rank tests are shown. ∗ Indicates p-value <0.05. (D) Kaplan-Meier plot of post-relapse survival for iMBSHH upfront treatment groups: CSI +CTx, Focal +CTx, HD-CTx only, IVT-MTX only and SD-CTx only. At-risk tables (number censored in parentheses) and p-values from log-rank tests are shown. CTx = Chemotherapy. RTx = Radiotherapy. CSI = Craniospinal irradiation. SD = Standard-dose. HD = High-dose. IVT-MTX = Intraventricular methotrexate. Supplementary Figure 3: (A) Kaplan-Meier plot of progression-free survival (PFS) and overall survival (OS) for iMBSHH patients treated with upfront CSI and CTx. (B) Kaplan-Meier plot of progression-free survival (PFS) and overall survival (OS) for iMBSHH patients treated with upfront Focal RTx and CTx. (C) Kaplan-Meier plot of progression-free survival (PFS) and overall survival (OS) for iMBSHH patients treated with upfront HD-CTx only. (D) Kaplan-Meier plot of progression-free survival (PFS) and overall survival (OS) for iMBSHH patients treated with upfront IVT-MTX only. (E) Kaplan-Meier plot of progression-free survival (PFS) and overall survival (OS) for iMBSHH patients treated with upfront SD-CTx only. At-risk tables are shown (number censored in parentheses). CTx = Chemotherapy. RTx = [file mmc1.pdf]
