# Supplementary material for: Therapy and biomarker dependent progression-free survival in infant sonic hedgehog medulloblastoma: a multi-national retrospective cohort study
Source: eClinicalMedicine. 2026 May 18;96:103913. doi: 10.1016/j.eclinm.2026.103913 (PMC13316349; doi:10.1016/j.eclinm.2026.103913)
Supplement: Supplementary Table S1 [file mmc2.pdf]

All DN/MBEN

<table>
<tr><th>Study Trial</th><th colspan="2">ACNS1221</th><th colspan="2" rowspan="2">Our study</th><th colspan="2">CCG 99703</th><th colspan="2">non trial
as per CCG 99703</th><th colspan="2">Headstart III</th><th colspan="2" rowspan="2">Our study</th><th colspan="2">HIT-2000</th><th colspan="2">non trial
as per HIT-2000</th><th colspan="2" rowspan="2">Our study</th></tr>
<tr><td>Publication</td><td colspan="2">Lafay-Cousin et al (2020)</td><td colspan="2">Cohen et al (2015)</td><td colspan="2">Lafay-Cousin et al (2016)</td><td colspan="2">Dhall et al (2020)</td><td colspan="4">Mynarek et al (2020)</td></tr>
<tr><td>Tx</td><td colspan="2">All SD</td><td colspan="2">SD</td><td colspan="2">All HD</td><td colspan="2">All HD</td><td colspan="2">All HD</td><td colspan="2">HD</td><td colspan="4">All HIT-SKK (IVT-MTX)</td><td colspan="2">IVT-MTX</td></tr>
<tr><td>n(DN/MBEN)</td><td colspan="2">25</td><td colspan="2">22</td><td colspan="2">14</td><td colspan="2">17</td><td colspan="2">27</td><td colspan="2">29</td><td colspan="2">42</td><td colspan="2">69</td><td colspan="2">66</td></tr>
<tr><td>Group</td><td colspan="2">All SHH</td><td colspan="2">SHH n=20/22</td><td colspan="2">N/A</td><td colspan="2">SHH n=14/14</td><td colspan="2">N/A</td><td colspan="2">SHH n=27/29</td><td colspan="2">SHH n =28/28</td><td colspan="2">All SHH</td><td colspan="2">All SHH</td></tr>
<tr><td>5 yr PFS</td><td colspan="2">53% (CI, 32-72%)
(2 yr PFS)</td><td colspan="2">57% (CI, 39-83%)</td><td colspan="2">All 79% ± 11%</td><td colspan="2">All 92 ± 7%</td><td colspan="2">All 89 ± 6%</td><td colspan="2">74% (CI, 58-93%)</td><td colspan="2">93% (CI, 85-100%)</td><td colspan="2">N/A</td><td colspan="2">73% (CI, 63-85%)</td></tr>
<tr><td>M status</td><td colspan="2">All M0</td><td>M0 n=13</td><td>M+ n=9</td><td>M0 = 13</td><td>M+ = 1</td><td>M0/M1 = 16</td><td>M2/M3 = 1</td><td>M0 = 15</td><td>M+ = 11</td><td>M0 n=19</td><td>M+ n=8</td><td colspan="4">All M0</td><td>M0 n=59</td><td>M+ n=7</td></tr>
<tr><td>5 yr PFS</td><td colspan="2">53% (CI, 32-72%) (2 yr PFS)</td><td>54%
(CI, 33-89%)</td><td>64%
(CI, 38-100%)</td><td>85% ± 10%</td><td>N/A</td><td>92 ± 8%</td><td>N/A</td><td>93 ± 6%</td><td>82 ± 12%</td><td>77%
(CI, 59-100%)</td><td>73%
(CI, 47-100%)</td><td colspan="4">93% (CI, 85-100%)</td><td>77%
(CI, 67-89%)</td><td>43%
(CI, 18-100%)</td></tr>
<tr><td>SHH 1 vs SHH 2</td><td>SHH 1 n=10</td><td>SHH 2 n=15</td><td>SHH 1 n=6</td><td>SHH 2 n=14</td><td colspan="2" rowspan="3">N/A</td><td colspan="2" rowspan="3">N/A</td><td colspan="2" rowspan="3">N/A</td><td>SHH 1 n=7</td><td>SHH 2 n=15</td><td>SHH 1 n=15</td><td>SHH 2 n= 13</td><td>SHH 1 n= 41</td><td>SHH 2 n=30</td><td>SHH 1 n=30</td><td>SHH 2 n=30</td></tr>
<tr><td>5 yr PFS</td><td>30%
(2 yr PFS)</td><td>67%
(2 yr PFS)</td><td>16%
(CI, 3-99%)
(2 yr PFS)</td><td>85%
(CI, 68-100%)
(2 yr PFS)</td><td>80%
(CI, 52-100%)</td><td>72%
(CI, 52-100%)</td><td>100%</td><td>84%
(CI, 67-100%)</td><td>62%
(CI, 48-81%)</td><td>83%
(CI, 90-98%)</td><td>63%
(CI, 48-83%)</td><td>89%
(CI, 79-100%)</td></tr>
<tr><td>Log ranktest</td><td colspan="2">ns</td><td colspan="2">p=0.007</td><td colspan="2">ns</td><td colspan="4">ns in individual or combined cohorts</td><td colspan="2">p=0.017</td></tr>
</table>

Supplementary Table 1: Clinical studies in DN/MBEN iMB
DN=Desmoplastic nodular. MBEN=Medulloblastoma with extensive nodularity. Tx=Therapy. SD=Standard dose chemotherapy. HD=High dose chemotherapy. IVT-MTX=Intraventricular methotrexate. M0=Non-metastatic disease. M+=Metastatic disease.
PFS=Progression free survival. CI=Confidence interval (95%). ns=not significant (p>0.05).
