# Supplementary material for: Therapy and biomarker dependent progression-free survival in infant sonic hedgehog medulloblastoma: a multi-national retrospective cohort study
Source: eClinicalMedicine. 2026 May 18;96:103913. doi: 10.1016/j.eclinm.2026.103913 (PMC13316349; doi:10.1016/j.eclinm.2026.103913)
Supplement: Supplementary Table S2 [file mmc3.pdf]

	MB_{SHH} total cohort (n=267)	MB_{SHH} survival cohort (n=226)	CSI + CTx (n=49)	Focal +CTx (n=25)	HD-CTx (n=29)	IVT-MTX (n=72)	SD-CTx (n=31)	p value (All treatment arms)	p value (HD-CTx vs IVT-MTX vs SD-CTX)
**Age at diagnosis (years)**									
<3.0y	199 (74.5%)	167 (73.9%)	16 (32.7%)	22 (88%)	26 (89.7%)	59 (81.9%)	30 (96.8%)		
3-5.0y	68 (25.5%)	59 (26.1%)	33 (67.3%)	3 (12%)	3 (10.3%)	13 (18.1%)	1 (3.2%)	**<0.001**	0.104
No data	0	0	0	0	0	0	0		
Median (range)	2 (0-4.94)	2 (0-4.94)	3.0 (0-4.73)	1.9 (0.5-3.3)	1.7 (0.2-3.0)	2.0 (0.5-4.9)	1.4 (0.2-3.0)		
**Sex**									
Male	141 (53.4%)	123 (54.7%)	21 (42.9%)	17 (68%)	15 (51.7%)	41 (56.9%)	18 (58.1%)		
Female	123 (46.6%)	102 (45.3%)	28 (57.1%)	8 (32%)	14 (48.3%)	31 (43.1%)	13 (41.9%)	0.2941	0.863
M:F ratio	1.1	1.2	0.8	2.1	1.1	1.3	1.4		
No data	3	1	0	0	0	0	0		
**Resection**									
GTR	173 (77.6%)	165 (77.1%)	33 (67.3%)	18 (75%)	25 (86.2%)	54 (75%)	24 (82.8%)		
STR	50 (22.4%)	49 (22.9%)	16 (32.7%)	6 (25%)	4 (13.8%)	18 (25%)	5 (17.2%)	0.4214	0.433
No data	44	12	0	1	0	0	2		
**Pathology**									
CLA	36 (15.3%)	29 (13.6%)	11 (15.1%)	4 (10.5%)	1 (1.8%)	3 (2.2%)	7 (14.3%)		
DN	128 (54.2%)	115 (53.7%)	23 (31.5%)	13 (34.2%)	16 (29.1%)	36 (26.1%)	13 (26.5%)		
MBEN	58 (24.6%)	57 (26.6%)	5 (6.8%)	3 (7.9%)	11 (20%)	30 (21.7%)	7 (14.3%)	**0.001**	**0.041**
LCA	14 (5.9%)	13 (6.1%)	6 (8.2%)	2 (5.3%)	0 (0%)	3 (2.2%)	2 (4.1%)		
DN/MBEN	186 (78.8%)	172 (80.4%)	28 (38.4%)	16 (42.1%)	27 (49.1%)	66 (47.8%)	20 (40.8%)		
No data	31	12	4	3	1	0	2		
**Metastatic disease**									
M0	185 (80.8%)	177 (81.2%)	37 (78.7%)	21 (87.5%)	20 (74.1%)	64 (88.9%)	21 (70%)		
M+	44 (19.2%)	41 (18.8%)	10 (21.3%)	3 (12.5%)	7 (25.9%)	8 (11.1%)	9 (30%)	0.134	**0.045**
No data	38	8	2	1	2	0	1		
***MYC* amplification**									
Yes	4 (1.6%)	4 (2.0%)	1 (2.2%)	0 (0%)	1 (4.2%)	0 (0%)	1 (3.3%)		
No	241 (98.4%)	200 (98.0%)	45 (97.8%)	24 (100%)	23 (95.8%)	60 (100%)	29 (96.7%)	-	-
No data	22	22	3	1	5	12	1		
***MYCN* amplification**									
Yes	16 (6.5%)	15 (7.4%)	7 (15.2%)	0 (0%)	1 (4.2%)	6 (10%)	1 (3.3%)		
No	229 (93.5%)	189 (92.6%)	39 (84.8%)	24 (100%)	23 (95.8%)	54 (90%)	29 (96.7%)	-	-
No data	22	22	3	1	5	12	1		
**SHH subgroup**									
SHH-1	94 (36.7%)	81 (37.7%)	8 (18.6%)	14 (58.3%)	8 (28.6%)	32 (45.1%)	11 (36.7%)		
SHH-2	124 (48.4%)	101 (47.0%)	18 (41.9%)	7 (29.2%)	15 (53.6%)	33 (46.5%)	19 (63.3%)	<0.001	0.116
SHH-3	35 (13.7%)	31 (14.4%)	16 (37.2%)	3 (12.5%)	5 (17.9%)	5 (7%)	0 (0%)		
SHH-4	3 (1.2%)	2 (0.9%)	1 (2.3%)	0 (0%)	0 (0%)	1 (1.4%)	0 (0%)		
No data	11	11	6	1	1	1	1		
**RTx**									
RTx at diagnosis									
Yes	81 (37.3%)	74 (35.4%)	49 (100%)	25 (100%)	0 (0%)	0 (0%)	0 (0%)		
No	136 (62.7%)	135 (64.6%)	0 (0%)	0 (0%)	29 (100%)	72 (100%)	31 (100%)	-	-
No data	50	17	0	0	0	0	0		
RTx type at diagnosis									
Focal	26 (12.0%)	25 (12.0%)	0 (0%)	25 (100%)	0 (0%)	0 (0%)	0 (0%)		
CSI	54 (25.0%)	49 (23.4%)	49 (100%)	0 (0%)	0 (0%)	0 (0%)	0 (0%)	-	-
None	136 (63.0%)	135 (64.6%)	0 (0%)	0 (0%)	29 (100%)	72 (100%)	29 (100%)		
No data	51	17	0	0	0	0	0		
CSI dose* at diagnosis									
Low and standard dose RTx (>2	22 (10.4%)	19 (9.3%)	19 (42.2%)	0 (0%)	0 (0%)	0 (0%)	0 (0%)		
High dose RTx (>27Gy)	27 (12.8%)	26 (12.7%)	26 (57.8%)	0 (0%)	0 (0%)	0 (0%)	0 (0%)	-	-
None	162 (76.8%)	160 (78.0%)	0 (0%)	25 (100%)	29 (100%)	72 (100%)	31 (100%)		
No data	56	21	4	0	0	0	0		
**CTx**									
CTx at diagnosis									
Yes	218 (97.3%)	210 (97.2%)	46 (93.9%)	25 (100%)	29 (100%)	72 (100%)	31 (100%)		
No	6 (2.7%)	6 (2.8%)	3 (6.1%)	0 (0%)	0 (0%)	0 (0%)	0 (0%)	-	-
No data	43	10	0	0	0	0	0		
Methotrexate administration									
Intranvenous (IV-MTX)	30 (17.8%)	30 (17.9%)	7 (20.6%)	3 (15%)	6 (42.9%)	0 (0%)	9 (39.1%)		
Intraventricular (IVT-MTX)	6 (3.6%)	6 (3.6%)	2 (5.9%)	0 (0%)	0 (0%)	4 (5.6%)	0 (0%)		
IV + IVT MTX	70 (41.4%)	69 (41.1%)	1 (2.9%)	0 (0%)	0 (0%)	68 (94.4%)	0 (0%)	-	-
None	63 (37.3%)	63 (37.5%)	24 (70.6%)	17 (85%)	8 (57.1%)	0 (0%)	14 (60.9%)		
No data	98	58	15	5	15	0	8		
CTx dose at diagnosis									
Standard dose CTx (SD)	100 (45.9%)	95 (45.2%)	36 (78.3%)	22 (88%)	0 (0%)	0 (0%)	31 (100%)		
High dose CTx (HD)	42 (19.3%)	40 (19.0%)	7 (15.2%)	3 (12%)	29 (100%)	0 (0%)	0 (0%)	-	-
IVT-MTX	76 (34.9%)	75 (35.7%)	3 (6.5%)	0 (0%)	0 (0%)	72 (100%)	0 (0%)		
No data	49	16	3	0	0	0	0		
CTx only at diagnosis (Radiotherapy naïve)									
Yes	133 (97.8%)	132 (97.8%)	-	-	29 (100%)	72 (100%)	31 (100%)		
No	3 (2.2%)	3 (2.2%)	-	-	-	-	-	-	-
No data	0	0	-	-	-	-	-		
**Median follow-up time (years)**									
OS (range)	3.93 (0.08-28.92)	4.00 (0.08-28.90)	6.12 (0.33-22.25)	4.77 (0.8-14.52)	3.93 (0.28-17.90)	3.00 (0.42-12.50)	4.57 (0.08-18.91)	-	-
**5 year PFS and OS**									
PFS (95% CI)	0.69 (0.63-0.76)	0.69 (0.63-0.76)	0.74 (0.62-0.88)	0.58 (0.41-0.82)	0.73 (0.58-0.93)	0.73 (0.63-0.84)	0.48 (0.33-0.71)	0.066	0.036
OS (95% CI)	0.81 (0.76-0.87)	0.82 (0.77-0.88)	0.82 (0.71-0.94)	0.71 (0.55-0.92)	0.89 (0.77-1.00)	0.86 (0.77-0.96)	0.72 (0.57-0.91)	0.2	0.086

Supplementary Table 2: iMB_{SHH} cohort table.

Cohort demographic and clinical data, including treatment protocols.

M=Male. F=Female. GTR=Gross total resection. STR=Subtotal resection. . CLA=Classic histology. LCA=Large cell/anaplastic histology. DN=Desmoplastic nodular. MBEN=Medulloblastoma with extensive nodularity. M0=Non-metastatic disease. M+=Metastatic disease. CSI=Craniospinal irradiation. RTx=Radiotherapy. IV=Intravenous.
IVT-MTX=Intraventricular methotrexate. CTx=Chemotherapy. PFS=Progression free survival. OS=Overall survival. *CSI dose: low and standard dose CSI <27Gy; high dose CSI ≥ 27Gy.
P values from chi-squared and log-rank tests are shown. Fisher's exact test was used in instances where the cell values are below 5.
